# A Nanorobotics-Based Approach of Breast Cancer in the Nanotechnology Era

**DOI:** 10.3390/ijms25094981

**Published:** 2024-05-02

**Authors:** Anca-Narcisa Neagu, Taniya Jayaweera, Krishan Weraduwage, Costel C. Darie

**Affiliations:** 1 Laboratory of Animal Histology, Faculty of Biology, “Alexandru Ioan Cuza” University of Iași, Carol I bvd. 20A, 700505 Iasi, Romania; aneagu@uaic.ro; 2 Biochemistry & Proteomics Laboratories, Department of Chemistry and Biochemistry, Clarkson University, Potsdam, NY 13699-5810, USA; jayawetm@clarkson.edu (T.J.); weraduk@clarkson.edu (K.W.)

**Keywords:** breast cancer (BC), nanorobots, nanobiomedicine, nano-theranostics

## Abstract

We are living in an era of advanced nanoscience and nanotechnology. Numerous nanomaterials, culminating in nanorobots, have demonstrated ingenious applications in biomedicine, including breast cancer (BC) nano-theranostics. To solve the complicated problem of BC heterogeneity, non-targeted drug distribution, invasive diagnostics or surgery, resistance to classic onco-therapies and real-time monitoring of tumors, nanorobots are designed to perform multiple tasks at a small scale, even at the organelles or molecular level. Over the last few years, most nanorobots have been bioengineered as biomimetic and biocompatible nano(bio)structures, resembling different organisms and cells, such as urchin, spider, octopus, fish, spermatozoon, flagellar bacterium or helicoidal cyanobacterium. In this review, readers will be able to deepen their knowledge of the structure, behavior and role of several types of nanorobots, among other nanomaterials, in BC theranostics. We summarized here the characteristics of many functionalized nanodevices designed to counteract the main neoplastic hallmark features of BC, from sustaining proliferation and evading anti-growth signaling and resisting programmed cell death to inducing angiogenesis, activating invasion and metastasis, preventing genomic instability, avoiding immune destruction and deregulating autophagy. Most of these nanorobots function as targeted and self-propelled smart nano-carriers or nano-drug delivery systems (nano-DDSs), enhancing the efficiency and safety of chemo-, radio- or photodynamic therapy, or the current imagistic techniques used in BC diagnosis. Most of these nanorobots have been tested in vitro, using various BC cell lines, as well as in vivo, mainly based on mice models. We are still waiting for nanorobots that are low-cost, as well as for a wider transition of these favorable effects from laboratory to clinical practice.

## 1. Introduction

The present time is widely recognized as an era of nanoscience and nanotechnology [1]. Nanoscience is the extensive research of the nano-sized structures, known as nanomaterials, ranging between 1 and 100 nm, while nanotechnology utilizes the nanoscience-derived nanomaterials for a wide variety of practical applications [2]. With the rapid development of nanotechnology, innumerable nanomaterials have been invented, fabricated and tested [3], and they have found applications in various fields of engineering, physical and chemical sciences, energy and the environment, as well as in biology and medicine [4], such as cell and molecular biology [5], biological separation, molecular imaging, biosensing and anti-cancer therapy [6,7]. Micro- and nanorobots, often biologically inspired, as in the case of biomimetic nanostructures, have defined the nano(bio)robotics era, performing multiple tasks at small scale for earlier and minimally invasive diagnostics, targeting drug delivery and localized minimally-invasive microsurgery [8]. Also, intelligent nanorobots are biocompatible, can be biodegradable, navigate throughout different environments, detect and kill cancer cells in the blood or other tissues and assure the most appropriate doses of drugs [9]. 

Ingeniously, over the last few years, human creativity has allowed for multiple types and forms of micro- and nanorobots to be designed that can be used for cancer theranostics, including BC. Thus, we can include here biohybrid flagellar polymer-based nanoswimmers that resemble spermatozoa [10], other types of helix-like nanorobots/nanorobot helices loaded with anti-cancer chemotherapeutic agents, which mimic a bacterial flagellum movement [11], urchin-head/drug-loaded hollow tail nanorobots with sharp nanospikes that resemble the surface of some urchins [12], octopus-like robots that combine both the morphology of the octopus sucker and the chemical proprieties of the mussel foot for improvement of adhesion in wet conditions [13], DNA nanorobots/walkers and molecular spiders, such as DNA nanorobots that target lysosomal degradation of BC specific proteins, which can “walk”, turn and stop, across the landscape, changing their shape, depending on pH changes in living organisms or that can be tagged with fluorescent molecules [11], magnetically propelled fish-like nanoswimmers/artificial nanofishes that imitate the body and caudal fin propulsion swimming mechanism displayed by fish for active drug delivery in various biomedical applications [14] or intelligent nanorobots that use fish swarm strategy for tumor targeting [15]. Moreover, Yang and Reif (2023) described social DNA nanorobots that execute a variety of operations, in the form of an engineered nano-dance between individual nanorobots, that have also developed novel collective behaviors [16]. These nanorobots’ collective activities were be inspired by sociobiology-based studies of these behaviors within social insects communities [16]. In this context, the emerging nanosafety concept must handle the unique characteristics and specific behavior of nanomaterials to understand their potential toxicity/immunotoxicity and epi-geno-toxicity [17]. To exemplify this, the use of hazardous materials and UV light in nanorobots as well as the loss of targeted control, which could make them unpredictable and uncontrollable, should be addressed in future risk-related studies of nanorobots [11]. Consequently, many authors directed their attention to green synthesis of materials, promoting environmentally-friendly approaches for the sustainable development of nanorobotics and nanotechnology [11,18]. Moreover, the fabrication, testing, improvement and clinical implementation of nanorobots also have the high limitation of development costs [19]. Thus, the laboratory synthesis of DNA nanorobots exceeds 1000 USD at the nanomole scale, while an adult human can require 300 nanomoles, with a cost of 300,000 USD/dose [20]. The use of more cost-effective nanomaterials could reduce the cost of the production of nanodevices used for cancer therapy [21]. 

Nowadays, nanotechnologies are applied in BC theranostics [22], including modern nanotherapies based on nanoscale drug delivery systems (nano-DDS) for targeting, multifunctionality [23] and nanodiagnostics, as the application of nanobiotechnology in molecular diagnosis that became an essential step for personalized and precise oncomedicine [24]. It is known that earlier detection, accurate BC diagnosis and targeted therapies are crucial for applying effective and personalized treatments [25]. Advances in efficient nano-DDSs design are becoming more and more suitable for differentiating between healthy and cancer cells to inhibit or kill cancer cells, by increasing the local concentration or efficiency of drug molecules exclusively in the targeted organelles, cells, tissues or organs, minimizing systemic cytotoxicity or undesirable adverse effects [26] and assuring the effective and prolonged release of chemotherapeutic drugs, active gene fragments and immune enhancing factors [27]. Nanoparticles (NPs) [28], liposomes [29], micelles, nanogels, dendrimers, exosomes and other nano-DDS for doxorubicin (DOX) delivery, among other drugs used in chemotherapy, can be used in the treatment of various BC subtypes, such as triple negative breast cancer (TNBC), which is known to be highly aggressive and recurrent [30]. 

In this review, readers will be able to deepen their knowledge of the structure, behavior and role of nanorobots, among other nanomaterials, in BC theranostic. We have summarized here the characteristics of several functionalized nanodevices that have been designed to counteract the main neoplastic hallmark features of BC by sustaining proliferative pathways [31,32], evading growth suppressors/evasion of anti-growth signaling [12], resisting programmed cell death [33,34,35,36,37,38,39,40,41,42], inducing angiogenesis (neovascularization) [43,44,45,46], activating invasion and metastasis [47,48,49], preventing genomic instability, mutation, mitosis/cell cycle deregulation [50], evading/avoiding immune destruction [51] and deregulating autophagy [52,53]. 

## 2. Nano-“Magic Bullets” in BC Theranostics

**Figure 1:**
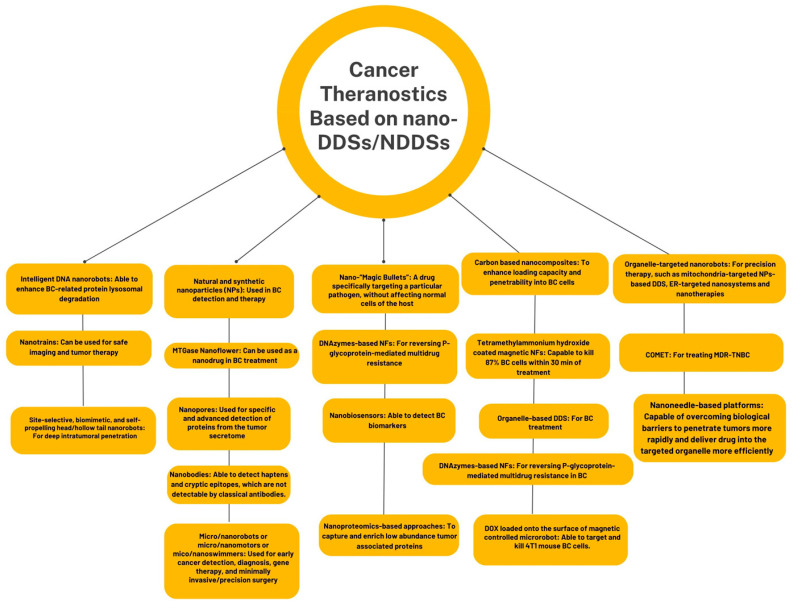
Diversity and function of nanorobots in BC theranostics.

In 1907, Paul Ehrlich, whose work was related to the development of chemotherapy and specific targeted treatment concepts [54], introduced the term “magic bullet” as a drug specifically targeting a particular pathogen, without affecting the normal cells of the host, anticipating the era of the development of site-specific therapies for cancer treatment [55,56]. Today, the use of nanomedicine and nano(bio)technology in the BC field involves handling modern and challenging variants of Ehrlich’s “magic bullet”, which can be considered as nano-“magic bullets” that are able to perform multiple and targeted functions and tasks, such as different types of nanorobots/nanobots/nanovehicles/nanomachines/nanomotors/nanodevices or nanosubmarines/nanosubs [21,57], nanotrains [58], nanostars [59], enzymatic, magnetic or DNAzyme based nanoflowers/nanoclusters [60,61,62], urchin-head/hollow tail nanorobots with sharp nanospikes [12], nanospheres [63], nanocubes [64], nanorods [65], nanoneedles [66], nanotubes [67], worm-like nanocrystal micelles [68], nanoshells [43], nanosponges and nanokillers [47], nanoknives [69], nanoballons [70], nanozymes [71], nano-snowflakes [25], nanobubbles [72,73], nanoemulsions [74], nanobodies [75], nanobiosensors [76], nanopores [77], nanocages [33], nanotraps [34],or nanogenerators [78] (Figure 1).

Undoubtedly, the use of nanoparticulate-based platforms has provide more and more advantages to the BC field including great biocompatibility and biodistibution, multifunctionality, the ability to overcome biological barriers and bioaccumulate in multiple tumor sites, even in the nucleus and specific organelles, reduced degradation and prolonged blood circulation time, passive or active targeting, effective drug delivery and low side effects [3]. Consequently, both natural and synthetic nanoparticles (NPs) have been extensively used in BC detection and therapy [79]. Nanobiosensors are able to detect BC biomarkers, such as specific genes, micro RNA, proteins, CTCs, BC cell lines, exosomes and exosome-derived biomarkers [76]. Nanopore-based single-molecule detection can actually be used for the specific and advanced detection of proteins from the tumor secretome, which can intravasate in blood or extravasate in different body fluids, using nanobody-functionalized nanopore sensors [77,80]. Recently, Zhang et al. (2023) reported a label- and amplification-free detection platform, using an engineered nanopore of the bacterial virus phi29 DNA-packaging motor with biomarker galectin 3 (GAL3) and the Thomsen–Friedenreich (TF) binding peptide, to detect basal levels of these biomarkers from nipple aspirate fluid (NAF) samples from BC patients at the single-molecule level [77]. Furthermore, today, various omics fields explore the patient’s body at the molecular level, for the discovery of potential biomarkers of disease, preferably in liquid, minimally or non-invasive biopsies [81]. Thus, nanoproteomics-based approaches allow for the integration of both nanotechnology and proteomics to capture and enrich low-abundance tumor-associated proteins from human serum or other body fluids as well, using NPs with functionalized surface combined with the analysis of proteoforms by mass spectrometry (MS) [82]. Zhang et al. (2023) developed a nanoproteomics approach, designing a novel aptamer-modified nano-sized metallic–organic framework (NMOFs-Apt)-based nanomaterial for serum epidermal growth factor receptor (EGFR) family proteins enrichment and the quantitative analysis of both HER2 and EGFR/HER1, using liquid chromatography–tandem mass spectrometry (LC-MS/MS)-based targeted proteomics [83]. 

Nanoneedle-based platforms, functionalized with hyaluronic acid as the main extracellular matrix (ECM) component, and loaded with chemotherapeutic agents, are capable of overcoming biological barriers to penetrate tumors more rapidly and deliver drugs into the targeted organelles more efficiently [66]. Bioactive and biocompatible carbon-based nanocomposites have been ingeniously bioengineered as smart nano-carriers to enhance their loading capacity and penetrability into BC cells for BC therapy [67]. Parenteral administration of nanoemulsions can be principally applied to deliver chemotherapeutic substances to the BC TME [74]. Bioorthogonal nanozymes, obtained from the encapsulation of transition metal catalysts into nanomaterials, act as “drug factories” that remain present at the tumor site at least one week after a direct injection, continuously converting the non-toxic molecules in the prodrug into active drugs at the injection site [71]. Nanobodies, a novel class of antibodies used for immunohistochemical detection that were discovered in camelids, are able to detect haptens and cryptic epitopes, which are not detectable by classical antibodies [75]. Under a microscope, nanoflowers (NFs), a distinctive subtype of nanomaterials, resemble flowers with a branched aspect and tailored petal structure that have a high surface-to-volume ratio [60]. Fatima et al. (2021) reported the antitumor effect of an enzymatic microbial transglutaminase nanoflower (MTGase NF) on MCF7 BC cells, based on the ability of MTGase to act as a molecular glue to allow for the development of a uniform shape in petals in this NF-like material that can be used as a nanodrug in BC treatment [60]. In addition, Patel et al. (2023) reported the synthesis of monodispersed teramethylammonium hydroxide-coated (Mn_0.5_Zn_0.5_Fe_2_O_4_) magnetic NFs, also called nanoclusters, that are capable of killing almost 87% of the MDA-MB-231 BC cells within 30 min of treatment, due to their magnetic fluid hyperthermia dispersion generated by the magnetic NPs in the presence of an external alternating magnetic field [61]. Moreover, Liang et al. (2022) constructed DNAzymes-based NFs with satisfactory biocompatibility and gene silencing ability for reversing P-glycoprotein-mediated multidrug resistance in BC [62]. 

As well as nanoflowers, nanotrains are programmable DNA-based nanostructures that can be used for safe imaging and tumor therapy [84]. Thus, Xu et al. (2019) designed a self-assembled, aptamer-conjugated DNA nanotrain (TA6NT-AKTin-DOX), which consists of a C44 aptamer TA6 and DNA building blocks conjugated with an AKT inhibitor peptide (AKTin), both individually and doxorubicin (DOX)-loaded [58]. This combinatorial nanotrain can reverse the drug resistance of BC stem cells (BCSCs), including apoptosis resistance and ABC transporters overexpression, via the AKT signaling pathway in MCF7 cells and xenografting BCSCs into nude mice [58].

Futuristic nanosubmarines/nanomachines/nanomotors/nanorobots, as pre-programmed/controlled or self-propelled bodies that can autonomous travel/navigate anywhere in the body, are able to “sense-and-act” through detecting substances and decontaminating the environment [85], to find and kill cancerous cells, perform transport, distribution and targeted drug delivery and microsurgery, and they were described quite a long time ago [57]. Today, it is known that cellular nanorobots, including DNA-nanorobots, are designed to recognize many different types of cancer cells, working at the cellular and subcellular levels, and utilizing their “nanoscale intelligence” to “sense, signal, respond and process”, as the in case of surgical oncology and cellular repair nanorobots [86]. To expand upon this, respirocytes were thought to be nanorobots mimicking red blood cells to carry oxygen and carbon dioxide [87], while microbivores and clottocytes can be used as engineered surrogates for phagocytes or other white blood cells and platelets, respectively [88]. Nanorobots or nanomaterials with an organelle-level resolution, also known as organelle-targeted nanorobots/nanomaterials, are viewed as the next generation of nanomedical devices for precision therapy [89], such as mitochondria-targeted NPs-based DDS [90], endoplasmic reticulum (ER)-targeted nanosystems and nanotherapies [91], Combinatorial Organelle Mitochondrial Endoplasmic Reticulum Therapy (COMET) as a novel nanomedicine for treating multidrug resistant triple negative breast cancer (MDR-TNBC) [92] or intelligent DNA nanorobots that are able to enhance BC-related protein lysosomal degradation [52]. In addition, with the development of biomimetic nanoscience, organelles, such as lysosomes, mitochondria, naturally occurring extracellular vesicles (EVs) [93] and lipid droplets, were used as drug carriers that could be incorporated in organelle-based DDS for BC treatment, having a good biocompatibility, high drug loading efficiency, modifiability and ability to enhance intracellular and intercellular communication due to specific surface biomarkers, as well as having the therapeutic effects of drugs [94]. Moreover, site-selective, biomimetic and self-propelling head/hollow tail nanorobots were designed for efficient extravasation from blood vessels, active cellular internalization and remodeling of the dense tumor stromal microenvironments for deep intratumoral penetration, suppressing tumor growth in a bone metastasis female mouse model of TNBC [12]. 

Micro/nanorobots (MNRs) or micro/nanomotors, also known as micro/nanoswimmers, can mainly act as “motile-cancer targeting” micro- or NDDS [95] that have a combination of load, transport and deliver-based abilities [96], and are also being used for early cancer detection and diagnosis, gene therapy and minimally invasive/precision surgery [21]. Microrobots have submillimeter dimensions [96], while nanorobots/nanobots are nano-devices that carry out precision tasks at the nanoscale level (1–100 nm) [86]. Chemicals, in the case of chemically-powered MNRs, external fields (optical, optoelectronic, acoustic, and magnetic), for external-field-powered MNRs, or motile cells, in biological or biohybrid MNRs, can be used for autonomous movement, micromanipulation and navigation in different biological microenvironments of micro/nanoswimmers [97]. MNRs can cross many biological barriers, such as the blood–brain barrier (BBB) and dense extracellular matrixes (ECMs) [95], and also become true nanosubmarines in the bloodstream [21]. 

Different types of single-cell microrobots, such as cell-based microrobots, bacteria-based microrobots and algae-based microrobots [27], have been largely developed in recent years [98] to exploit the natural mobility and other features of cells/organisms for the transport and biodistribution of different drugs [99]. Cell-based microrobots, such as erythrocytes, sperm cells [100], leukocytes and other motile cells driven microrobots, are considered effective for targeted drug delivery, due to their escape mechanisms and biocompatibility [98]. Magnetic bacteria-based bio-hybrid microrobots are a widely used living material in the field of magnetically driven diagnostics [101] as well as for targeted cancer therapy [102]. Microalgae, due to their high biocompatibility, low costs, large active surface area, strong anti-cancer drug and NPs absorption capability, phototaxis, oxygen production and high-speed of propulsion, can be successfully used as oxygenators, micro- or nanoswimmers and ideal carriers for efficient drug loading and precise targeted DDS [99,103]. Thus, Zhong et al. (2020) reported the efficacy of a biodegradable microalgae-based carrier, *Spirulina platensis*, for the targeted delivery of doxorubicin and non-invasive fluorescence imaging-guided therapy on lung metastasis of 4T1 mouse BC cells [27]. 

Magnetically-driven/powered biological/natural and bio-hybrid MNRs are composed of main body and magnetic material [104]. Thus, the main body material can be made from different single-cell types and naturally or artificially loaded with the magnetic material, such as commonly used iron oxide nanoparticles (NPs) [104]. In the case of biomicrorobots/biobots, magnetosomes are intracellular structures containing natural occurring iron-rich magnetic NPs [105]. Synthetized by certain strains of magnetotactic bacteria (MTB) [26,106], magnetosomes are membrane-enclosed magnetic/iron oxide (Fe_3_O_4_) NPs, which can be included in magnetosome-based DDS [26]. Song et al. (2022) reported a DOX loaded onto the surface of magnetic controlled microrobot via electrostatic interactions that exhibited pH-responsive release behavior and was capable of polarizing macrophages into the anti-tumor phenotype to target and kill 4T1 mouse BC cells [107].

## 3. Breast Cancer Hallmark Features

In 2000, Hanahan and Weinberg emphasized the first generation of cancer hallmarks, consisting of sustaining proliferative signaling, evading growth suppressors, resisting cell death, enabling replicative immortality, inducing angiogenesis and activating invasion and metastasis [108]. In 2011, these authors formulated the next generation of hallmarks of cancer, adding genome instability, tumor-promoting inflammation, reprogramming energy metabolism and evading immune destruction [109]. In 2022, Hanahan added another four additional emerging hallmarks and enabling characteristics of cancer: unlocking phenotypic plasticity, non-mutational epigenetic reprogramming, polymorphic microbiomes and senescent cells [110]. Moreover, it is now established that the components of the tumor microenvironment (TME) contribute to different cancer hallmarks [111], while the molecular mechanisms involved in autophagy also have a role in the hallmarks of cancer [112]. Studying the research effort involved in elucidating each of these hallmarks and their relevance for BC, Saha et al. (2021) placed invasion and metastasis as the central hallmark, suggesting that this hallmark is the most highly explored, followed by sustaining proliferative signaling in the primary tumor, inducing angiogenesis, resisting apoptosis, enabling replicative immortality, evading growth suppressors, genomic instability, reprogramming energy metabolism, evading immune destruction and tumor-promoting inflammation [113]. 

**Figure 2:**
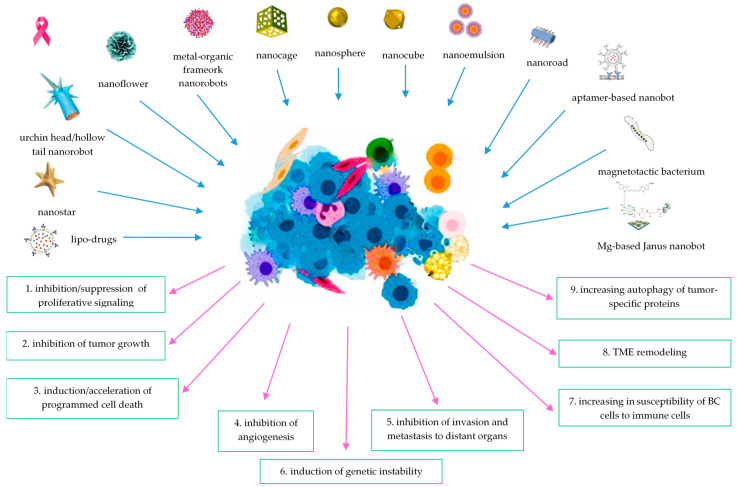
BC hallmarks targeted by nanorobots.

Nanotechnologies have been widely applied in oncological research through multiple strategies to target and alleviate these hallmarks, from automatic nanorobots that induce mitochondria-mediated apoptosis and mitochondrial deregulation to improve the anticancer effects and suppression of cancer metastasis [89] to nanotechnology-based metabolic reprogramming strategies for enhanced tumor immunotherapies [114], or to self-propelling nanosized robots in blood, for CTCs capture [48]. The multiple roles of several types of nanorobots, among other nanomaterials, in BC therapy and diagnosis are summarized in Figure 2. 

## 4. Theranostic Roles of Nanomaterials against BC Hallmarks

### 4.1. Nanomaterials Used against Sustained Proliferative Signaling in BC Cells

Abnormal proliferation is a hallmark of cancer development and progression, so cancer treatments target and kill cells that have a high level of proliferation and regeneration [115]. Tumor necrosis factor alpha (TNF-α) is a pro-inflammatory cytokine involved in cell proliferation, differentiation and apoptosis, and also enhances the effects of chemo- and radiotherapy against BC cells [116]. However, TNF-α plays a dual function, acquiring both pro- and antitumor actions [36]. Jawad et al. (2021) designed a DDS involving PEGylated gold nanoparticles (GNPs) loaded with TNF-α and demonstrated the anti-proliferative effects of TNF-α against an Iraqi AMJ13 BC cell line, which also resulted resulting in apoptosis and mitochondrial damage [31]. Circular RNAs (circRNAs) act as microRNA sponges and regulate gene expression as they are related to the hallmarks of cancer-sustaining signaling pathways, including invasion, recurrence, metastasis, apoptosis, ferroptosis and treatment resistance [117]. Zhou et al. (2023) showed that delivering CREBZF mRNA NPs can inhibit BC proliferation and promote apoptosis in both BC tissues and cell lines [32]. These authors have proposed a new circRNA, called circPAPD4, whose expression is low in BC tissues and cells, that acts as a sponge by binding miRNA-1269a to increase the expression of CREBZF, a transcription factor that inhibits the activation of STAT3 pathway, leading to a reduction in cell cycle progression [32].

### 4.2. Nanomaterials for Avoiding Evading Growth Suppressors/Evasion of Anti-Growth Signaling in BC

The evasion of anti-growth signaling is an essential feature of cancer cells [118]. It is known that gold nanoparticles (AuNPs) inhibit tumor growth via different mechanisms, such as mitochondrial apoptosis, OS and metabolic stress, with decreased glycolysis in a c-Myc-dependent manner [119]. Moreover, gold clusters can prevent BC bone metastasis and secondary osteolysis, suppressing the migration, invasion and colony formation of MDA-MB-231 BC cells [120]. Furthermore, ROS-responsive galactosylated NPs functionalized with DOX (DOX@NPs) inhibited or suppressed the growth of TNBC cells (4T1) in vitro and in vivo, triggering apoptosis and cell cycle arrest [28]. It is known that non-targeted liposomes are currently used for BC treatment, while the targeted liposomes are currently progressing through clinical trials [121]. Thus, lipo-drugs for combinatorial chemotherapies, including the DNA synthesis inhibitor gemcitabine (GC) and the microtubule polymerization inhibitor mertansine (DM1), like EGFR antibody-liposomes-GC/DM1, have been reported to inhibit the growth of MDA-MB-231 and MDA-MB-469 TNBC cell lines and drug resistance in vitro and in vivo [29]. Recently, Yu et al. (2024) developed a multifunctional NPs-based platform (CSA-ss-Ce6/CSSC), consisting of chondroitin sulfate (CSA) for the targeted delivery of chlorin e6 (Ce6) and DOX against TNBC cell lines 4T1 and MDA-MB-231 as well as for 4T1-bearing Balb/c in a mice model [122]. Furthermore, this chemo-photodynamic therapy based on CSSC-D NPs enhanced the generation of ROS under NIR, exerting cytotoxic effects and tumor growth inhibition. 

To suppress TNBC growth in a bone metastasis female mouse model and different subcutaneous tumor models, Yan et al. (2023) designed site-selective, self-propelling and biomimetic head/hollow tail nanorobots that enable the efficient remodeling of the dense tumor stromal microenvironment (TSM) by decreasing stromal cell viability and leading to the denaturation of the ECM, to assure a deep intratumoral penetration [12]. These nanorobots are suggestively called asymmetric urchin-head/hollow tail nanostructures (UHHTNs—AuNS@SiO_2_ core/shell NPs), due to their numerous surface-located nanospikes that resemble the surface of some urchins. UHHTN consists of near-infrared (NIR)-absorbed AuNS half-coated with a SiO_2_ shell in the head region and a large open hollow tail connected to the half shell that enables encapsulation of stimuli responsive phase-change materials and DOX drugs, which can be triggered by NIR irradiation, due the photothermal effect of AuNS which increases the temperature for on-demand delivery [12]. 

### 4.3. Anti-Resisting Cell Death Nanomaterials

Inducing apoptosis, a well-characterized form of programmed cell death, is an important strategy for controlling excessive BC cell proliferation by extrinsic and intrinsic apoptotic pathways [123]. Anaerobic bacteria, such as magnetotactic bacteria (MTB) that form magnetosomes as intracellular nanometer-scale magnetic crystals, are able to preferentially replicate and accumulate in the hypoxic regions of solid tumors [35]. Moreover, MTB can also lead to increased apoptosis in human BC cells [35]. Exploiting these bacterial abilities, Menghini et al. (2023) showed that a particular species of MTB, *Magnetospirillum magneticum* (AMB-1 strain), known as a magnetically responsive organism and a carrier [102], interferes with proliferation and leads to increased apoptosis of MDA-MB-231 BC cell linse [35]. Cadmium telluride quantum dots (QDs), such as CdTe QDs, high-yield CdTe QDs, and CdTe/CdS core/shell QDs, also induced apoptosis in MDA-MB-468 and MCF7 BC cell lines [38]. Chaudhari et al. (2022) showed that surface-modified metallic NPs could solve the problem of microRNAs (miRNAs) delivery [37]. Thus, taking account that tumor suppressor miR-206 was reported as significantly downregulated in the luminal A BC subtype, these authors employed a PEG capped AuNPs system for the delivery of an miR-206 mimic to induce apoptosis in MCF7 BC cells through the downregulation of neurogenic locus notch homolog protein 3 (NOTCH3) [37]. Moreover, silver nanoparticles-coated ethyl cellulose (AgNPs-EC) also induced apoptosis in MCF7 BC cells, acting as an inhibitor of TNF-α production [36]. 

Cancer cells exhibit aberrant redox homeostasis and are characterized by a relative vulnerability to oxidative stress (OS), defined as an excess of reactive oxygen (ROS) and nitrogen species (RNS) [124]. Consequently, the first-line immune response to tumor cell hyperproliferation is an increased local production of ROS/RNS nearby cancer cells due to white blood cells’ innate immune response that generates an inflammatory landscape, followed by an increase in cancer cell apoptosis and cell cycle arrest [39]. Seyedi et al. (2022) proposed a human immune cell-stimulated anti-BC nanorobot (hisABC-NB) as an intelligent and safe antitumor agent for human BC therapy [39]. This type of nanorobot was produced by conjugating the mouse-derived inducible nitric oxide synthase (iNOS) and human-originated myeloperoxidase (MPO) on the folate-linked chitosan-coated Fe_3_O_4_ NPs functionalized with folic acid as the BC cells detector [39]. The hisABC-NB significantly reduced the number of MCF7 BC cells by inducing apoptosis and cell cycle arrest compared to the normal MCF10 cell type, and also emphasizing MRI contrast in the tumor region [39]. Protein nanocages, formed by the self-assembly of protein units, have been studied as potential nano-carriers for biomedical applications [125]. Thus, Ji et al. (2022) developed a low toxic, pH-sensitive, and high-efficiency targeting DDS, called Cur@HFn, using recombinant human heavy chain apoferritin (HFn), a hollow cage-like molecule, loaded with curcumin (Cur), a polyphenol flavonoid with anticancer proprieties in Chinese medicine [33]. HFn particles are able to easily cross biological barriers, have a deep tissue penetration ability and pH-sensitive self-assembly proprieties, being recognized by transferrin receptor 1 (TfR1), which is overexpressed in several human BCs, including the murine BC 4T1 and human MDA-MB-231 BC cell lines. TfR1 promotes the internalization of these particles, so that Cur@HFn induces a strong cytotoxicity in BC cell models, low systemic toxicity, high in vitro therapeutic effects, enhanced intracellular ROS in cancer cells, ROS-mediated DNA damage and cell apoptosis [33]. 

Mitochondria are the primary source of ROS derived from mitochondrial respiration and are also considered as a core organelle involved in ferroptosis [126], an iron-dependent programmed form of cell death caused by lipid peroxidation [127,128], due to the dysregulation of iron homeostasis in iron-rich tumors, like BC, that are particularly sensitive to ferroptosis-targeted drugs [40]. The localized image-guided ferroptosis in cancer nanomedicine can be performed through the use of remotely controllable magnetic nano-carriers [129]. Moreover, Yu et al. (2024) proposed a high intensity focused ultrasound (HIFU)-driven nanomotor (NP-G/P) based on PEGylated poly (lactic-co-glycolic acid) (PLGA) NPs loaded with perfluorooctyl bromide (PFOB) to endow responsiveness to HIFU forces for propulsion and on-demand drug release that activates ferroptosis-mediated antitumor immunity in primary and metastatic TNBC models, resulting in significant tumor regression and metastasis prevention [40].

Wu et al. (2021) elaborated upon this and fabricated a biocompatible free radical nanogenerator with NIR II laser-induced synergistic nitric oxide (NO) and alkyl radical release properties, named P(IR/BNN6/AIPH)@Lip-RGD [41]. This nanogenerator includes IR 1061, an NIR II molecule, BNN6, an NO donor, and AIPH, an alkyl radical initiator, which was firstly encapsulated in a natural lecithin and further functionalized by the 1,2-distearoyl-sn-glycero-3-phosphoethanolamine—polyethylene glycol–arginine–glycine–aspartic acid (DSPE-PEG-RGD) to have a specific tumor targeting ability. Thus, this nanogenerator can significantly inhibit the growth of breast tumors upon laser exposure, inducing cancer cell apoptosis via a mitochondria-mediated apoptotic pathway and the generation of mitochondrial ROS and the downregulation of Bcl-2 protein expression, accelerating cytochrome c release and triggering a cascade of apoptosis-related proteins caspase-3 and caspase-9 [41].

Recently, apoptosis strategies based on mitochondrial Ca^2+^ overload and the use of Ca^2+^ nanogenerators in BC treatment have attracted attention [42]. Thus, Wang et al. (2024) constructed a multimodal Ca^2+^ nano-modulator that combined the effects of photothermal therapy (PTT), chemotherapy and mitochondrial Ca^2+^ overload to inhibit BC development [42]. Technically, this nano-modulator encapsulated curcumin (Cur) and indocyanine green (ICG) into CaCO^3−^ NPs, crosslinking sodium alginate (SA) on their surface. Functionally, this SCCI nano-modulator, or SA/Cur@CaCO^3−^ICG, induces large amounts of ROS, followed by tumor cell apoptosis, or directly kills tumor cells, reducing the mitochondrial membrane potential and downregulating ATP production by accumulating large amounts of Ca^2+^ and having an acidic pH [42]. Furthermore, Guo et al. (2024) designed and fabricated a Ca^2+^/Cu^2+^ dual-ion nanotrap to avoid cell apoptosis evasion by synchronously inducing both apoptosis and paraptosis, an alternative cell death pathway characterized by endoplasmic reticulum and/or mitochondrial swelling and cytoplasmic vacuolization [130] for BC treatment [34]. Thus, these authors used a Cu^2+^–tannic acid metal phenolic network that was embedded onto the amorphous calcium carbonate NP’s surface, followed by mDSPE-PEG/lipid capping to form the disulfiram (DSF)-loaded Ca^2+^/Cu^2+^ dual-ion nanotrap. This nanotrap is internalized by endocytosis in BC cells where it suffers acid-dependent biodegradation in the lysosomes for the simultaneous release of Ca^2+^, Cu^2+^ and DSF. Consequently, the released Ca^2+^ can cause calcium overload in mitochondria, followed by mitochondrial dysfunction, and leads to hydrogen peroxide overexpression and cell paraptosis, while Cu^2+^ ultimately leads to BC cell apoptosis [34]. Furthermore, Peng et al. (2023) reported a self-powered metal–organic framework (MOF)-based nanorobot with favorable biocompatibility and biodegradation abilities, called ZIF-67@DOX-TPP, which is capable of active mitochondria-targeted drug delivery, and is prepared by encapsulating doxorubicin-tri phenylphosphonium (DOX-TPP) with mitochondriotropic behavior inside a zeolitic imidazolate framework (ZIF-67) NPs [89]. Thus, the ZIF-67@DOX-TPP nanorobot induces mitochondria-mediated apoptosis and mitochondrial dysregulation, improving the in vitro antitumor effects and the suppression of cancer metastasis, which have also been evaluated in vivo, using a BC model/lung metastasis model [89]. Thus, it was demonstrated that the mitochondria play a key role in carcinogenesis, BC cell proliferation, invasion, apoptosis, tumor metabolism and chemoresistance [131].

### 4.4. Nanomaterials Promoting Anti-Angiogenic Effects

Hypoxia in TME is a characteristic hallmark of cancer that can transcriptionally activate genes that encode proteins that promote primary tumor vascularization/angiogenesis/neovascularization, following disturbances to the hypoxia-inducible factor (HIF) signaling pathway [132]. Yang et al. (2016), using MCF7 BC cells, showed that silver nanoparticles (AgNPs) can inhibit angiogenesis in vitro by disrupting the(HIF) signaling pathway and downregulating the vascular endothelial growth factor-A (VEGF-A) and glucose transporter 1 (GLUT1) transmembrane proteins, both known HIF target genes that can adapt to hypoxic environments [44,133,134]. Guo et al. (2016) engineered a TNBC-targeted and anti-angiogenic liposomal small interfering (siRNA) delivery system, named ICAM-Lcn2-LP, that binds to the intercellular adhesion molecule-1 (ICAM-1) from MDA-MB-231 cells [45]. ICAM-Lcn2-LPs downregulate lipocalin 2 (Lcn2), which is known as a promising therapeutic agent and a potential diagnostic biomarker for BC, due to its ability to promote BC progression by stimulating the EMT in BC cells and enhancing angiogenesis. Also, Lcn2 knockdown led to a significant reduction in VEGF from BC cells that led to reduced angiogenesis both in vivo and in vitro [45]. Sun et al. (2020) designed and developed SCMNPs, consisting of saikosaponin D, a triterpene saponin derived from *Bupleurum chinense*, and loaded them with macrophage-mimicking biomimetic DDS/NPs by coating them with a camouflaging macrophage-derived membrane, to form a shell hybridized with T7 peptide on the surface of a core made of poly (lactic-co-glycolic acid) NPs [46]. SCMNPs inhibited tumor growth and metastasis in BC in vivo and in vitro through VEGFR, AKT and ERK related to the angiogenic pathway [46]. Nunes et al. (2019) engineered hybrid anti-HER2 gold nanoshells (GNs) coated with PEG polymers and conjugated them to rabbit anti-human HER2 polyclonal antibodies for the purposes of photothermal therapy to overcome trastuzumab resistance in HER2-overexpressing BC xenograft models [43]. The core of GNs includes Fe_3_O_4_/SiO_2_ NPs functionalized with a poly (vinylimidazole-co-silane) polymer (PVIS) to bind small gold nuclei to the silica surface [43]. Thus, these multifunctional GNs are characterized by an anti-angiogenic and pro-apoptotic effect, leading to the inhibition of tumor growth/proliferation [43].

**Figure 3:**
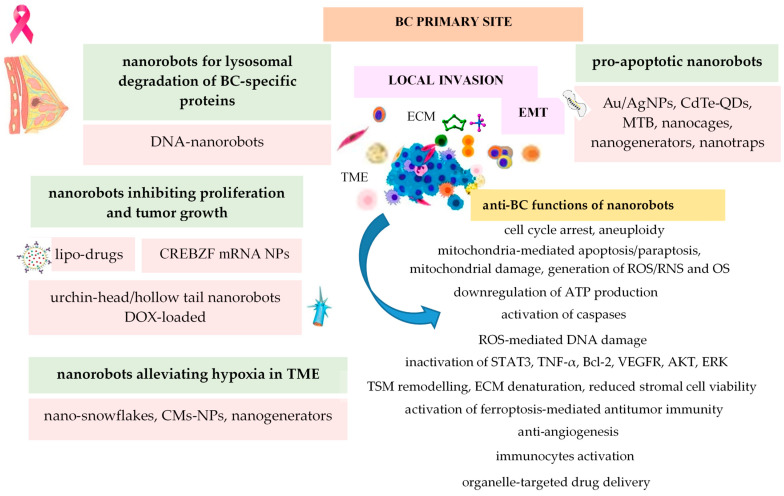
Anti-tumor functions of nanorobots at primary site of BC.

The most important anti-tumor functions that could be exerted by various engineered nanorobots at BC primary site are synthetized in Figure 3. 

### 4.5. Anti-Invasion and Anti-Metastatic Nanomaterials

Nanosponges (NSs) are 3D mesh-like/porous nanostructures that encapsulate and carry a wide variety of small drug molecules, increasing the biosolubilization and bioavailability of both hydrophilic and hydrophobic drugs [135]. It was demonstrated that nanosponge-based delivery systems may be superior to other DDSs because they can provide a high specificity in drug delivery and controlled/prolonged/sustained drug release, biocompatibility and degradability [136,137]. Moreover, 3D printing and eco-friendly technologies can be involved in the development of novel nanosponge-based systems for biomedical applications [137]. Tiwari et al. (2022) summarized several biomedical applications of nanosponges in cancer, as enzyme and biocatalyst carriers and poison absorbents, and for solubility enhancement, enzyme immobilization and oxygen delivery [136]. 

Immune cell membrane-based biomimetic nano-carriers have an increasing therapeutic efficacy against cancer metastasis through immune evasion, prolonged circulation, high tumor bioaccumulation and immunosuppression of the TME [138]. It is known that neutrophils can be used as carriers for cancer nanotherapeutics such as liposomes, magnetite NPs, and PEGylated poly (lactic-co-glycolic acid) (PLGA) [139], so that neutrophil membrane-coated NPs are able to keep the antigenic exterior and associated membrane functions of the host cells, making them ideal decoys for neutrophil-targeted biological molecules [140]. Moreover, the platelet membrane (PM) can be integrated into PM-cloaked NPs (PM@NPs) that increase the biocompatibility of DDS and reduce adverse reactions to drugs [141]. Ye et al. (2020) prepared doxorubicin (DOX) and indocyanine green (ICG) co-loading gold nanocages (AuNCs) for combination chemical–photothermal therapy and developed bionic platelets and neutrophil hybrid cell membrane (PNM)-camouflaging AuNCs [47]. Thus, nanosponges/nanokillers combined with photothermal/chemotherapy become have more effective in capturing and clearing CTCs, neutralizing migrating tumor-derived exosomes, activating the innate immune system and inhibiting BC metastasis in 4T1 xenograft and orthotopic breast tumor-bearing mice [47]. Furthermore, cytotoxic T lymphocyte (CTL)-inspired nanovesicles (MPVs) with a cell membrane-derived shell that camouflages gelatin nanogel cores loaded with methylene blue (MB) and cisplatin (Pt) generate a triple combination therapy, producing chemotherapeutical effects generated by Pt, hyperthermia upon laser irradiation and enabling photothermal imaging and high tumor penetration [49]. In addition, these combinatorial therapy kills 4T1 BC cells, resulting in primary tumor regression and a strong inhibition of pulmonary metastasis [49]. 

**Figure 4:**
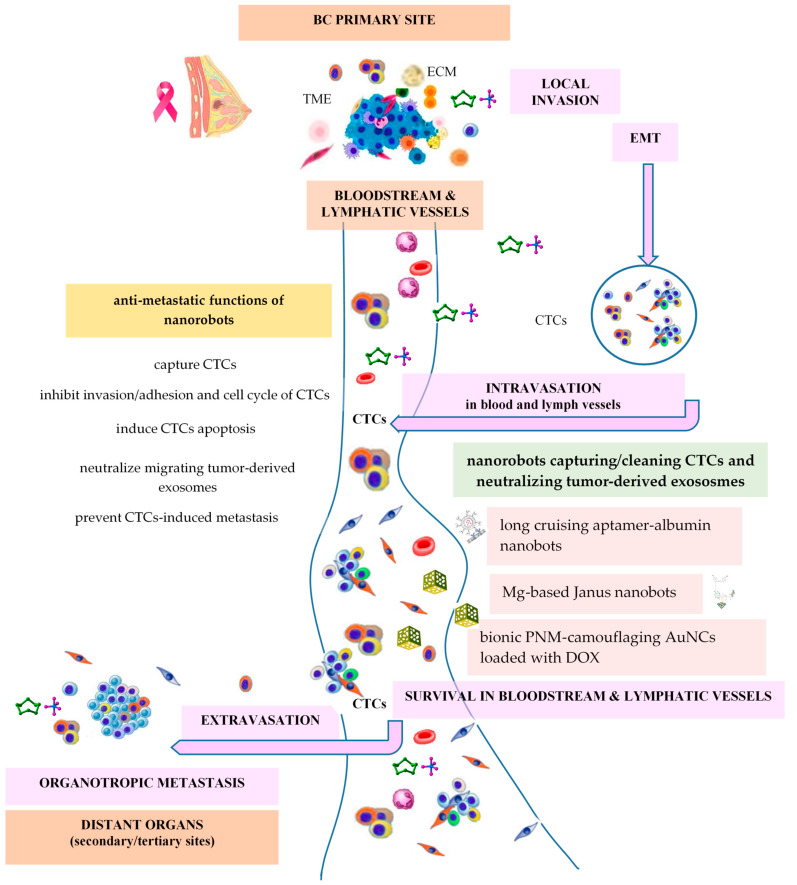
Anti-metastatic functions of nanorobots.

A self-propelled nanorobot with autonomous motion, based on the hydrogen bubbles produced by spontaneous a Mg–water reaction, has been reported by Wavhale et al. (2021) for the selective and rapid capture of CTCs [142]. This Mg-Fe_3_O_4_-GSH-G4-Cy5-Ab/Tf (called Janus MFN) nanobot was prepared by fabricating a hemispherical shell of Fe_3_O_4_ on Mg NPs selectively loaded with an anti-epithelial cell adhesion molecule (EpCAM) monoclonal antibody (Ab)/transferrin (Tf) for targeting MCF7 BC cells, a dye, cyanine 5 NHS (Cy5) for particle fluorescent labelling, a fourth generation (G4) dendrimer for multiple conjugation and a glutathione (GSH) linker. For a similar purpose, Wang et al. (2023) developed a long cruising and intelligent aptamer (AP)-albumin nanorobot able to capture and restrain CTCs by conjugation of CTC-targeting circulating trivalent aptamers (CTA) with human serum albumin (HSA), resulting in CTA-HSA nanorobots that are able to circulate for longer in the blood, with an increased probability of collision and the ability to capture more CTCs [48]. Anti-metastatic functions of nanorobots are illustrated in Figure 4.

### 4.6. Genomic Instability, Mutations, Mitosis/Cell Cycle Deregulation

Genomic instability (GI) is one hallmark feature of most cancer cells that is characterized by alterations in the cell cycle checkpoints, DNA repair machinery, mitotic checkpoints and telomere maintenance [143]. Thus, GI leads to karyotypic abnormalities and aneuploidy, increasing intratumoral heterogeneity [144], principally due to altered dynamics in the spindle-assembly checkpoint (SAC) that assures the high-fidelity separation of genetic material by informing the cell cycle machinery of putative errors in the interaction of chromosomes with spindle microtublules [145]. NPs made of the natural/biocompatible polymer chitosan may induce changes in cell morphology and the cellular junctions, and reduce motility, proving an anti-cancer protective effect [50]. Olmos et al. (2019) studied the potential sensitizing effect of chitosan-based nanoparticles (CS-NPs) treated with reversine on MCF7 BC cells exposed to X-ray irradiation [50]. Reversine was reported as an anticancer agent that acts as a selective inhibitor of mitotic kinase monopolar spindle 1 (MPS1), also triggering apoptotic cell death by decreasing anti-apoptotic proteins Bcl-XL and Mcl-1, increasing pro-apoptotic proteins and activating caspase-3 activity [146]. Moreover, reversine activates autophagy via the AKT signaling pathway, and upregulates hypoxia-inducible factor 1-alpha (HIF-1α) and glucose transporter 1 (GLUT1), followed by a reduction in glucose uptake and energy production in cancer cells [146]. To conclude this section, CS-NPs can affect mitosis and cell viability and sensitize MCF7 BC cells to X-ray irradiation [50].

### 4.7. Nanomaterials Targeting Evading/Avoiding Immune Destruction

BC cells can escape from the body’s immune response through multiple mechanisms of immune evasion, such as the modulation of TME and the modification of surface antigens [147]. Kang et al. (2015) showed that a mica NP (STB-HO), an alluminosilicate mineral, possesses anticancer and immunostimulatory effects, increasing the susceptibility of MCF7 BC cells to immune cells from their TME, stimulating the immunocytes to eliminate cancer cells, and reducing tumor growth in an MCF7 xenograft model [51]. Wang et al. (2024) showed that TME-resident macrophages promote tumor cell immune escape, EMT and invasion during the initial steps of cancer progression, so targeting M2-like tumor-associated macrophages (TAMs) can stop tumor growth, metastasis and drug resistance [148]. For this purpose, d’Avanzo et al. (2021) prepared LinTT1 peptide-functionalized liposomes that are able to interact with oncogenic M2 primary human macrophages, enhancing the cytotoxic effects of DOX and sorafenib co-loaded inside these liposomes on 2D and 3D BC cellular models [149]. Thus, LinTT1 peptide targets the p32 protein that is overexpressed by BC cells and TAMs in the hypoxic core-tumor area, where other nano-DDSs are not able to act [149]. Moreover, targeted, stimuli-responsive, self-assembled and therapeutic peptide-based nanosystems are used to enhance the effects of photodynamic therapy [150]. 

### 4.8. Nanomaterials Targeting Intratumoral/TME Hypoxia

Almost 90% of solid cancers have hypoxic TME a key hallmark, with low values of partial pressure of oxygen (pO_2_ < 10 mm Hg/1.5% O_2_) compared to 160 mm Hg/21% O_2_ in the ambient air or 14–65 mm Hg/2–9% O_2_ in different organs [151,152]. The BC hypoxic environment reduces oxygen-dependent free radical generation, whereas the overexpression of glutathione (GSH) in BC cells reduces the impact of free radical generation [78]. Consequently, it is well known that hypoxia mediates the pathobiological behavior of cancer cells, increasing their rapid proliferation, aggressiveness and invasiveness, and also reducing the sensitivity of tumors to chemotherapy, radiotherapy, photodynamic therapy (PDT) and immunotherapy [152,153]. To counteract the hypoxia related effects, nanotechnological strategies based on NP carriers and bio-carriers can increase the oxygen generation or delivery into hypoxic TME, improving the delivery of drug molecules and the efficacy of radiotherapy, as well as the infiltration of innate immune cells, enhancing the effects of immunotherapy [152]. Nanorobots also can resolve tumor hypoxia [21]. The ability of anaerobic bacteria to preferentially accumulate in the hypoxic areas of solid tumors can be exploited for the improvement of the antineoplastic mechanisms in bacterial cancer therapy [35]. Thus, the drug-loaded magneto-aerotactic bacteria of the strain MC-1 have been investigated as therapeutic nanorobots in cancer therapy, due to their capacity to be guided through their microaerophilic behavior by using an oxygen concentration that decreases towards the hypoxic tumoral area [154].

PDT is a non-invasive therapeutic approach characterized by oxygen dependence that can limit its therapeutic efficacy in solid tumors, so numerous strategies have explored the creation of new and proper photosensitizers with a higher photodynamic conversion efficiency that could decrease tumor hypoxia to fuel the generation of ROS [155]. Lv et al. (2022) developed a multimodal contrast agent that can be used for magnetic resonance imaging (MRI) and photoacoustic imaging (PAI), called the nano-snowflake probe (UMC—USPIO@MnO_2_@Ce6) for oxygen-enhanced photodynamic therapy (PDT), using a honeycomb-like MnO_2_ to co-load chlorin e6 (Ce6 as a photosensitizer) and ultra-small superparamagnetic iron oxide NPs (USPIO, T1–T2 double contrast agent) [25]. Thus, UMCs aggregate to the tumor region, promote the decomposition of H_2_O_2_ to O_2_, degrade and trigger the exposure of the photosensitizer to oxygen, accelerating the production of ROS during PDT. Furthermore, UMC enhances the therapeutic effects of Ce6 for PTD under laser (660 nm) irradiation, stimulating the inhibition of tumor growth, and effective anti-tumor therapy [25]. To improve hypoxia at tumor sites and enhance the efficacy of hypoxia-limited PDT therapy in triple negative breast cancer (TNBC), Fang et al. (2021) developed a cancer cell membrane-coated/biomimetic oxygen-delivery nanoprobe, called cancer cell membrane-coated human serum albumin-indocyanine green-doped perfluorotributylamine/perfluorocarbon (CCm-HAS-ICG-PFTBA) [156]. This biomimetic oxygen delivery nanoprobe enhanced the therapeutic efficacy of PDT and contains highly biocompatible ingredients, such as a perfluorotributylamine (PFTBA) core, which could dissolve a large amount of oxygen, and a cancer cell membrane coating, which enables homologous targeting and immune evasion, with potential for clinical translation [156]. Following the same hypothesis and to enhance the efficiency of radiotherapy (RT), Gao et al. (2017) have developed a nanoscale system called PFC@PLGA-RBCM NPs that does not require additional stimuli, using a biomimetic red blood cell membrane (RBCM) to envelop a perfluorocarbon (PFC) core incapsulated in poly (lactic-co-glycolic acid) (PLGA) [157]. The PFC core dissolves a large amount of oxygen and delivers it in the tumor, while the RBCM coating enables the prolonged circulation of NPs in blood [157]. 

Recently, alkyl radicals have been introduced into BC therapy due to their oxygen-independent generation properties [41]. Thus, Si et al. (2023) proposed a non-toxic, oxygen-independent free radical nanogenerator to enhance BC therapy, CuS/AIPH@BSA–copper monosulfide/2,2′-azabis(2-imidazoline) dihydrochloride@bovine serum albumin, that encapsulates an alkyl radical initiator, AIPH, within hollow mesoporous CuS NPs [78]. Functionally, AIPH was released and decomposed to generate alkyl radicals in hypoxic BC with the photothermal conversion effect of cooper monosulfide under near-infrared (NIR) laser irradiation. CuS consumes high levels of GSH in tumor cells, enhancing free radical treatment with anticancer in vivo and in vitro efficacy [78]. Before that, Wu et al. (2021) produced a biocompatible free radical nanogenerator with NIR II laser-induced simultaneous nitric oxide (NO) and alkyl radical release property for BC therapy [41]. 

### 4.9. Nanomedicine for Deregulating Autophagy Modulation

Autophagy is known as a self-catalytic and self-protective program that is responsible for the degradation and recycling of abnormal or unneeded cellular proteins or other macromolecules and damaged organelles to maintain proteostasis and cellular homeostasis, and provide energy, so that activated autophagy enables cancer cells to survive and rapidly adapt and evade most therapies [112,158]. However, activated autophagy can also promote cancer cell death through the excessive degradation of cellular components [159]. Generally, autophagy involves the formation of autophagosomes that sequester cytoplasmic material and deliver it to lysosomal compartments for degradation by lysosomal hydrolases [112]. 

In this context, NPs can be used as nano-carriers, but they may also have the ability to alter the signaling pathway networks and molecules involved in autophagy regulation [159]. Tang et al. (2017) reviewed many common nanomaterials that can induce autophagy: gold NPs, quantum dots (QD), titanium dioxide NPs, zinc oxide NPs, nano rare earth oxides, fullerene, fullerenol and carbon nanotubes [3]. Recently, Lewinska et al. (2024) emphasized the role of surface-modified magnetic nanoparticles, such as iron-oxide/magnetite (Fe_3_O_4_ NPs), against chemotherapy-induced drug-resistant senescent BC cells, i.e., Hs _57_8T, BT-20, MDA-MB-468, and MDA-MB-175-VII lines, demonstrating their hyperthermia and OS-mediated anticancer effects [53]. Thus, in etoposide-stimulated non-senescent and senescent BC cells, glucosamine-based amorphous carbon coated NPs with reductive activity (Fe_3_O_4_@aC) caused an increase in the levels of autophagic (BECN1, LC3B), proinflammatory (NF-κB, IL-6, and IL-8), antioxidant (FOXO3a, SOD1, and GPX4) and cell-cycle-inhibitory (p21, p27, and p57) proteins, nucleolar stress and apoptotic cell death, in parallel with a decrease in ROS production [53]. The roles of other types of metal oxide NPs have been also demonstrated, such as copper oxide NPs (CuO NPs) that induces autophagy as a survival mechanism against CuO NP-mediated toxicity in an MCF7 BC cell line, while the inhibition of autophagy induces apoptosis [160]. 

Generally, DNA robots are able to recognize different types of cancer cells [86], meaning that intelligent/smart DNA nanorobots show great promise for nanomedicine due to their potential to improve the antitumor efficacy of nano-drug delivery systems for precision anticancer therapy [52]. Human epidermal growth factor receptor 2 (HER2) is a tyrosine–protein kinase receptor, whose overexpression and *HER2/neu* gene amplification play essential roles in BC development and progression by inducing oncogenic pathways such as PI3K/AKT, so that the anti-HER2 drugs target and bind to the HER2 protein expressed on the cancer cell surface [161]. Thus, Ma et al. (2019) reported an intelligent DNA nanorobot composed of an anti-HER2 aptamer on a tetrahedral framework nuclei acid (HApt-tFNA); in vitro, this DNA nanorobot is able to form HER2-HApt-tFNA complexes, remove HER2 protein from the plasma membrane of HER2-overexpressing cells (human mammary gland adenocarcinoma SK-BR-3 cell line) by HER2-mediated endocytosis and enhance HER2 protein lysosomal degradation, inducing BC cells apoptosis and arrested cell growth [52]. As molecular mechanisms, the authors demonstrated the inhibition of downstream PI3K/AKT pathway when HER2 protein expression decreased in SK-BR-3 cell membrane under the action of this intelligent DNA nanorobot. 

The theranostic roles of nanomaterials against BC hallmarks are summarized in Table 1.

## 5. Conclusions

**Figure 5:**
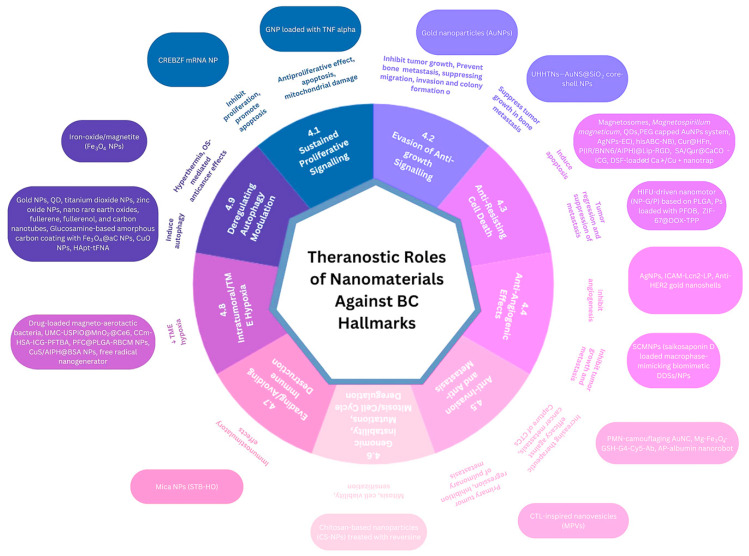
Theranostic roles of nanomaterials against BC.

Numerous nanomaterials, culminating in nanorobots, can be used in diverse applications in BC nano-theranostics. These ingeniously engineered nanorobots are thus designed to perform multiple tasks at a small scale, even at the organelles or molecular level, both in vitro, on various BC cell lines, as well as in vivo, on small animal models. Most nanorobots are biomimetic and biocompatible nano(bio)structures that are used as targeted nano-DDS, resembling different organisms and cells, such as an urchin, spider, octopus, fish, spermatozoon, flagellar bacterium or helicoidal cyanobacterium. In this review, we summarized multiple characteristics of many functionalized nanodevices designed to counteract the main neoplastic hallmark features of BC (Figure 5), such as sustaining proliferative signaling, evading growth suppressors/the evasion of anti-growth signaling, resisting programmed cell death, inducing angiogenesis, activating invasion and metastasis, genomic instability, mutation, mitosis/cell cycle deregulation, avoiding immune destruction and deregulating autophagy. These nanostructures can exert multiple anti-cancer behaviors by sustaining anti-proliferative effects, inducing and enhancing apoptosis, activating ferroptosis and paraptosis, suppressing tumor growth, inhibiting of angiogenesis, prevention and the inhibition of lung and bone metastasis, increasing the oxidative stress, inducing DNA-damage and proteolysis of dysregulated tumor-related proteins, alleviating hypoxia in TME, capturing and CTCs and neutralizing tumor-derived EVs involved in metastasis development, stimulating immune cells to eliminate BC cells, remodeling tumor stromal environments or sensitizing BC cells to uni- and multimodal antitumor therapy. Usually, most of these nanorobots function as targeted and self-propelled smart nano-carriers or nano-DDS, increasing the efficiency and safety of chemo-, radio- or photodynamic therapy, or of current imagistic techniques used in BC diagnosis. Furthermore, many pathways within BC cells, such as the STAT3, HIF signaling, PI3K/AKT or ERK pathways can be targeted, inhibited or disrupted by nanorobots that are used for BC nano-theranostics. We are still waiting for the transition of these favorable effects from in vitro research to preclinical trials on larger animals as well as for their adoption in future clinical practice.

## Figures and Tables

**Table 1:** Theranostic roles of nanomaterials against BC hallmarks.

Hallmarks of Cancer	Nanomaterials/Nanotherapies against BC Hallmarks	Functions	References
**1.** **Sustaining proliferative signaling**	Functionalized PEGylated gold NPs for TNF-α delivery (GNPs-TNFα).	Inhibition of the proliferation (AMJ13 BC cells), mitochondrial damage and apoptosis promotion.	[31]
	CREBZF mRNA NPs: circPAPD4-miR-1269a.	Inhibition of the proliferation and promotion of apoptosis by overexpression of CREBZF and inactivation of the STAT3 pathway; reduction in cell cycle progression, and the suppression of proliferation in vivo and in vitro (MCF7, SKBR-3, BT474, BT549, MDA-MB-468).	[32]
**2.** **Evading growth suppressors/evasion of anti-growth signaling**	Urchin-head/hollow tail nanorobots (UHHTNs-AuNS/SiO_2_ core/shell NPs): @-AuNS coated with a SiO_2_ shell.	TSM remodeling by reducing stromal cell viability and ECM denaturation; suppression of tumor growth in a bone metastasis female mouse model of TNBC and anticancer efficacy in different subcutaneous tumor models.	[12]
	ROS-responsive galactosylated NPs (DOX@NPs).	Inhibit/suppress the growth of TNBC (4T1) in vitro and in vivo, and trigger apoptosis and cell cycle arrest.	[28]
	Lipo-drugs for combined chemotherapy: antibody-liposome-GC/DM1 (EGFR mAb-lipo-drugs)	The combination of GC and DM1 inhibits TNBC growth in vitro and in vivo (MDA-MB-231, MDA-MB-468) and reduces drug resistance.	[29]
	Chondroitin sulfate-based nanoplatforms/NPs (CSA-ss-Ce6/CSSC and DOX-loaded CSSC)	Under NIR, CSSC-D enhanced ROS generation and cytotoxicity/growth tumor inhibition against TNBC cells (4T1, MDA-MB-231) and 4T1-bearing Balb/c mice model.	[122]
**3.** **Resisting programmed cell death**	**Magnetotactic bacteria (MTB):** *Magnetospirillum magneticum* (AMB-1)	Magnetically targeted bacterial BC therapy, leading to increased apoptosis, and interfere with the proliferation of MDA-MB-231 BC cells.	[35]
	Silver **NPs**-coated ethyl cellulose (AgNPs-EC).	Induce apoptosis in MCF7 BC cells; act as inhibitors of TNF-α production.	[36]
	Functionalized PEG capped gold NPs (AuNPs) system for the miR-206 delivery/mimicry.	Induce apoptosis in MCF7 BC cells by NOTCH3 downregulation, arrest cell cycle, effective in luminal A subtype of BC treatment.	[37]
	cadmium telluride quantum dots: CdTe QDs, high yield CdTe QDs, CdTe/CdS core/shell QDs	induce apoptosis in MDA-MB-468 and MCF7 BC cell lines	[38]
	Nanocages for ROS-mediated apoptosis: Cur@HFn—hollow cage-like molecule of HFn loaded with curcumin (Cur) that decomposes in an acidic pH and reassembles to a neutral pH.	Strong cytotoxicity in BC cell models (murine 4T1 and MDA-MB-231 BC cell lines, 4T1 tumor-bearing mouse), low systemic toxicity, high in vitro therapeutic effects; enhances intracellular ROS in cancer cells, ROS-mediated DNA damage, BC cells apoptosis.	[33]
	Human immune cell stimulated anti-BC nanorobot (hisABC-NB): iNOS and MPO enzymes on the folate-linked chitosan-coated Fe_3_O_4_ NPs functionalized with folic acid as BC cells detector.	Reduced MCF7 by inducing ROS/RNS and OS, cancer cell apoptosis and cell cycle arrest; useful for MRI-mediated traceability.	[39]
	Nanorobots for ferroptosis-immunotherapy: HIFU-driven nanomotor/NP-G/P driven by HIFU- high intensity focused ultrasound-driven nanomotor (PLGA NPs loaded with PFOB).	Activates ferroptosis-mediated antitumor immunity in TNBC models, leading to tumor regression and metastasis prevention; HIFU induces cell stress, triggering the expression of ferroptosis-associated genes (HOX1, GST, SQSTM etc.).	[40]
	Free radical releasing nanogenerator for synergistic NO and alkyl radical therapy of BC: P(IR/BNN6/AIPH)@Lip-RGD.	Inhibits breast tumors growth, induces cancer cells apoptosis via a mitochondria-mediated apoptotic pathway and generation of mitochondrial ROS, downregulates Bcl-2, accelerate cytochrome c release and triggers a cascade of apoptosis-related caspase-3 and caspase-9.	[41]
	Ca^2+^ nanogenerator/nano-modulator: SA/Cur@CaCO^3-^ICG (SCCI)—curcumin (Cur) and indocyanine green (ICG) into CaCO^3-^ NPs, crosslinking sodium alginate (SA).	Induces large amounts of ROS followed by tumor cell apoptosis or directly kills tumor cells, reducing mitochondrial membrane potential and downregulating ATP production by producing large amounts of Ca ^2+^ and acidic pH.	[42]
	Ca^2+^/Cu^2+^ dual-ion nanotrap (disulfiram (DSF)-loaded amorphous calcium carbonate NPs) : Cu^2+^-tannic acid metal phenolic network embedded onto amorphous calcium carbonate NPs surface, followed by mDSPE-PEG/lipid capping.	Released Ca^2+^ causes mitochondrial calcium overload and H_2_O_2_ overexpression; Ca^2+^/ROS-associated mitochondrial dysfunction causes paraptosis cell death; released Cu^2+^ will ultimately induce cell apoptosis.	[34]
	ZIF-67@DOX-TPP nanorobot: mitochondriotropic DOX-triphenylphosphonium inside zeolitic imidazolate framework-67 NPs.	Designed for mitochondria-targeted drug delivery; ZIF-67 body decomposes H_2_O_2_ in tumor cells, induces mitochondria-mediated apoptosis and mitochondrial dysregulation and has in vitro and in vivo anticancer effects and suppresses cancer metastasis.	[89]
**4.** **Inducing angiogenesis (neovascularization)**	Anti-angiogenic **silver NPs** (AgNPs).	Inhibit angiogenesis in MCF7 BC cells through the disruption of the HIF signaling pathway and downregulation of HIF target genes (VEGF-A and GLUT1).	[44]
	Multifunctional gold nanoshells.	Inhibition of tumor growth/inhibition of proliferation due to an anti-angiogenic effect, and increased apoptosis; combined with photothermal therapy, can overcome trastuzumab resistance in HER2-overexpressing BC cells.	[43]
	Anti-angiogenic liposomal siRNA delivery system: ICAM-Lcn2-LPs.	Targets and binds to ICAM-1 from hTNBC MDA-MB-231 BC cells; induces Lcn2 knockdown, and reduces VEGF and angiogenesis in vitro and in vivo.	[45]
	Macrophage-mimicking NPs/DDSs (SCMNPs): saikosaponin D (SsD) loaded macrophage membrane hybridized with T7 peptide on the surface of PLGA NPs.	Inhibit VEGFR, AKT and ERK related to the angiogenic pathway, tumor growth and metastasis of BC cells in vitro and in vivo.	[46]
**5.** **Activating invasion and metastasis**	Anti-metastatic nanosponges/nanokillers: bionic PNM-camouflaging AuNCs loaded with DOX.	Combined with chemo and photothermal therapy, capture and clear CTCs, neutralize migrating tumor-derived exosomes, inhibit invasion and metastasis in 4T1 xenograft and orthotopic BC-bearing mice.	[47]
	Long cruising anti-metastatic aptamer-albumin nanobots: CTA-HSA—three hairpin-shape nucleic acid APs targeting EpCAM used to produce biostable CTA conjugated with HSA.	Cruise in blood longer, capture more CTCs, escape the immune clearance, inhibit invasion/adhesion and cell cycle of CTCs, induce CTCs apoptosis and prevent CTC- induced metastasis	[48]
	CTL-inspired nanovesicles (MPV) with a cell membrane-derived shell and MB and Pt loaded gelatin nanogel core.	Generate contrast for tumor photo-acoustic imaging, produce hypothermia upon laser irradiation, enabling photothermal imaging and deep tumor penetration; kill 4T1 BC cells, resulting in primary tumor regression and inhibition of pulmonary metastasis.	[49]
	Mg-based Janus nanobots (MFN): (Mg)-Fe_3_O_4_-based Magneto-Fluorescent Nanorobot-shell of Fe_3_O_4_ NPs with EpCAM antibody/transferrin for targeting CTCs, Cy5 for fluorescent labelling and G4 dendrimer for multiple conjugation and GSH linker (Mg-Fe_3_O_4_-GSH-G4-Cy5-Ab/Tf).	Self-propelled in blood, and capture CTCs (MCF7 cells) with high efficiency,	[142]
**6.** **Genomic instability, mutation, mitosis/cell cycle deregulation**	Chitosan-NPs (CS-NPs) treated with reversine and X-ray irradiation.	CS-NPs affect mitosis and cell viability and sensitize MCF7 BC cells to X-ray irradiation by passive or targeted bioaccumulation in cancer cells; reversine induces premature exit from mitosis, aneuploidy and cell death.	[50]
**7.** **Evading/avoiding immune destruction**	Mica NPs (STB-HO)	anticancer and immunostimulatory effect; increase susceptibility of MCF7 BC cells to immune cells and stimulate the immunocytes to eliminate BC cells	[51]
	LinTT1 peptide-functionalized liposomes loaded with DOX and sorafenib.	Targets p32 overexpressed by BC cells (MCF7, MDA-MB-231) and TAMs/oncogenic M2 macrophages in hypoxic area of tumor.	[149]
**8.** **TME hypoxia**	Multimodal nano-snowflakes: UMC (USPIO@MnO_2_@Ce6)—honeycomb-like MnO_2_ to load Ce6 as photosintetizer and ultrasmall superparamagnetic iron oxide NPs.	Used both for multimodal MRI/PAI-guided antitumor therapy, targeting intratumoral hypoxia; aggregates to the tumor region and promotes the decomposition of H_2_O_2_ to O_2_, enhancing the therapeutic effect of Ce6 for PDT under laser irradiation; inhibit tumor growth.	[25]
	Biomimetic nanoscale systems based on cell membranes (CMs)-coated NPs for high oxygen delivery: CCm-HSA-ICG-PFTBA—CCm-coated human serum albumin-indocyanine green-doped perfluorotributylamine/perfluorocarbon (PFC); PFC@PLGA-RBCM NPs– RBC-mimic system by encapsulating PFC within PLGA.	Targets tumor tissue, alleviates hypoxia in TME, enhances PDT efficacy in TNBC 4T1 BC cell line and 4T1 BALB/c mice xenografts.	[156,157]
	Oxygen-independent free-radical (alkyl radical) **nanogenerator:** CuS/AIPH@BSA—copper monosulfide NPs coated with BSA and loaded onto the alkyl radical initiator 2,2′-azabis(2-imidazoline) dihydrochloride.	Photothermal exposure accelerates BSA dissociation and exposes CuS, preventing GSH-mediated free radical consumption and providing oxygen-independent enhanced free radical treatment of hypoxic BC.	[78]
**9.** **Deregulating autophagy**	Carbon-coated iron oxide **NPs**: Fe_3_O_4_@aC NPs.	Hyperthermia and OS-mediated anticancer effects: decreased ROS production, increased level of antioxidant proteins, cell cycle inhibitors, proinflammatory and autophagic biomarkers, nucleolar stress, apototic cell death in drug-induced senescent BC cells (Hs _57_8T, BT-20, MDA-MB-468, MDA-MB-175-VII) and promoted reductive stress-mediated cytotoxicity in non-senescent BC cells.	[53]
	**DNA-based nanorobot** (HApt-tFNA): anti-HER2 AP on a tetrahedral framework nucleic acid.	HER2-HApt-tFNA induces HER2-mediated endocytosis, digestion in lysosomes, reduction of HER2 amount on the cell surfaces, inhibition of PI3K/AKT pathway, cell apoptosis, arrested cell growth.	[52]

## Abbreviations

Ab	Antibody
AgNPs	Silver nanoparticles
AIPH	Alkyl radical initiator
AP or Apt	Aptamer
AuNCs	Gold nanocages
BSA	Bovine serum albumin
CCm	Cancer cell membrane
Ce6	Chlorine 6
circRNA	Circular RNA
CS-NP	Chitosan nanoparticles
CTA	Circular trivalent aptamer
CTCs	Circulating tumor cells
CTL	Cytotoxic T lymphocyte
Cur	Curcumin
Cy5	Cyanine 5 NHS dye
DM1	Microtubule polymerization inhibitor mertansine
DOX	Doxorubicin
ECM	Extracellular matrix
EpCAM	Epithelial cell adhesion molecule
Fe_3_O_4_@aC	Glusosamine-based amorphous carbon coating magnetite NPs
Fe_3_O_4_@Dex	Magnetite NPs coated by dextran
GC	Gemcitabine
G4	Fourth generation
GLUT1	Glucose transporter 1
GSH	Reduced glutathione
GSH	Glutathione
GST	Glutathione S-transferase
HER2	Human epidermal growth factor receptor 2
H_2_O_2_	Hydrogen peroxide
HIF-1	Hypoxia-inducible factor-1
HFn	Human heavy chain apoferritin
HIFU	High intensity focused ultrasound
HOX1	Heme oxygenase
HSA	Human serum albumin
ICAM	Intercellular adhesion molecule-1
ICG	Indocyanine green
iNOS	Inducible nitric oxide synthase
Lcn2	Lipocalin 2
LPs	Liposomes
MB	Methylene blue
MCF7	Michigan Cancer Foundation-7
MPO	Myeloperoxidase
MRI	Magnetic resonance imaging
NIR	Near-infrared laser irradiation
NO	Nitric oxide
NOTCH3	Neurogenic locus notch homolog protein 3
NPs	Nanoparticles
NSs	Nanosponges
NSKs	Nanosponges/nanokillers
OS	Oxidative stress
PAI	Photoacoustic imaging
PDT	Photodynamic therapy
PEG	Poly(ethylene glycol)
PFC	Perfluorocarbon
PFOB	Perfluorooctyl bromide
PFTBA	Perfluorotributylamine
PI3K/AKT	Phosphoinositide 3-kinase/protein kinase B
PLGA	Poly (lactic-co-glycolic acid) NPs
PNM	Platelet and neutrophil hybrid cell membrane
Pt	Cisplatin
QDs	Quantum dots
RBC	Red blood cell
RNS	Reactive nitrogen species
ROS	Reactive oxygen species
siRNA	Small interfering RNA
SQSTM	Sequestosome
TAMs	Tumor associated macrophages
Tf	Transferrin
tFNA	Tetrahedral framework nucleic acid
TME	Tumor microenvironment
TNBC	Triple negative breast cancer
TSM	Tumor stromal microenvironment
USPIO	Ultrasmall superparamagnetic iron oxide
VEGF	Vascular endothelial growth factor

## References

[1] Ahire S.A., Bachhav A.A., Pawar T.B., Jagdale B.S., Patil A.V., Koli P.B. (2022). The Augmentation of nanotechnology era: A concise review on fundamental concepts of nanotechnology and applications in material science and technology. Results Chem..

[2] Bayda S., Adeel M., Tuccinardi T., Cordani M., Rizzolio F. (2020). The History of Nanoscience and Nanotechnology: From Chemical–Physical Applications to Nanomedicine. Molecules.

[3] Tang X., Loc W.S., Dong C., Matters G.L., Butler P.J., Kester M., Meyers C., Jiang Y., Adair J.H. (2017). The use of nanoparticulates to treat breast cancer. Nanomedicine.

[4] Wang G. (2023). Editorial: Nanomaterials for biology and medicine. Front. Chem..

[5] Wang E.C., Wang A.Z. (2013). Nanoparticles and their applications in cell and molecular biology. Integr. Biol..

[6] Pham S.H., Choi Y., Choi J. (2020). Stimuli-Responsive Nanomaterials for Application in Antitumor Therapy and Drug Delivery. Pharmaceutics.

[7] Awasthi A., Awasthi K.K., John P.J. (2021). Nanomaterials in Biology. Environ. Sci. Pollut. Res..

[8] Hu C., Pané S., Nelson B. (2018). Soft Micro- and Nanorobotics. Annu. Rev. Control Robot. Auton. Syst..

[9] Dolev S., Narayanan R.P., Rosenblit M. (2019). Design of nanorobots for exposing cancer cells. Nanotechnology.

[10] Chesnitskiy A.V., Gayduk A.E., Seleznev V.A., Prinz V.Y. (2022). Bio-Inspired Micro- and Nanorobotics Driven by Magnetic Field. Materials.

[11] Arvidsson R., Hansen S.F. (2020). Environmental and health risks of nanorobots: An early review. Environ. Sci. Nano.

[12] Yan M., Chen Q., Liu T., Li X., Pei P., Zhou L., Zhou S., Zhang R., Liang K., Dong J. (2023). Site-selective superassembly of biomimetic nanorobots enabling deep penetration into tumor with stiff stroma. Nat. Commun..

[13] Meloni G., Tricinci O., Degl’innocenti A., Mazzolai B. (2020). A protein-coated micro-sucker patch inspired by octopus for adhesion in wet conditions. Sci. Rep..

[14] Li T., Li J., Zhang H., Chang X., Song W., Hu Y., Shao G., Sandraz E., Zhang G., Li L. (2016). Magnetically Propelled Fish-Like Nanoswimmers. Small.

[15] Wen S., Sun Y., Chen S., Chen Y., Chen Y., Yao D., Nakano T. (2023). A Intelligent Nanorobots Fish Swarm Strategy for Tumor Targeting. Bio-Inspired Information and Communications Technologies.

[16] Yang M., Reif J., Jonoska N., Winfree E. (2023). Social DNA Nanorobots. Visions of DNA Nanotechnology at 40 for the Next 40.

[17] Lebre F., Chatterjee N., Costa S., Fernández-De-Gortari E., Lopes C., Meneses J., Ortiz L., Ribeiro A.R., Vilas-Boas V., Alfaro-Moreno E. (2022). Nanosafety: An Evolving Concept to Bring the Safest Possible Nanomaterials to Society and Environment. Nanomaterials.

[18] Gupta D., Boora A., Thakur A., Gupta T.K. (2023). Green and sustainable synthesis of nanomaterials: Recent advancements and limitations. Environ. Res..

[19] Giri G., Maddahi Y., Zareinia K. (2021). A Brief Review on Challenges in Design and Development of Nanorobots for Medical Applications. Appl. Sci..

[20] Hu Y. (2021). Self-Assembly of DNA Molecules: Towards DNA Nanorobots for Biomedical Applications. Think. Ski. Creativity.

[21] Kong X., Gao P., Wang J., Fang Y., Hwang K.C. (2023). Advances of medical nanorobots for future cancer treatments. J. Hematol. Oncol..

[22] Kumar P., Mangla B., Javed S., Ahsan W., Musyuni P., Sivadasan D., Alqahtani S.S., Aggarwal G. (2023). A review of nanomaterials from synthetic and natural molecules for prospective breast cancer nanotherapy. Front. Pharmacol..

[23] Ediriwickrema A., Saltzman W.M. (2015). Nanotherapy for Cancer: Targeting and Multifunctionality in the Future of Cancer Therapies. ACS Biomater. Sci. Eng..

[24] Alharbi K.K., Al-Sheikh Y.A. (2013). Role and implications of nanodiagnostics in the changing trends of clinical diagnosis. Saudi J. Biol. Sci..

[25] Lv Y., Kan J., Luo M., Yang C., Luo X., Lin X., Li H., Li X., Li Y., Yang C. (2022). Multifunctional Nanosnowflakes for T1-T2 Double-Contrast Enhanced MRI and PAI Guided Oxygen Self-Supplementing Effective Anti-Tumor Therapy. Int. J. Nanomed..

[26] Puri R., Arora V., Kabra A., Dureja H., Hamaal S. (2022). Magnetosomes: A Tool for Targeted Drug Delivery in the Management of Cancer. J. Nanomater..

[27] Zhong D., Zhang D., Xie T., Zhou M. (2020). Biodegradable Microalgae-Based Carriers for Targeted Delivery and Imaging-Guided Therapy toward Lung Metastasis of Breast Cancer. Small.

[28] Zhou J., Li K., Zang X., Xie Y., Song J., Chen X. (2023). ROS-responsive Galactosylated-nanoparticles with Doxorubicin Entrapment for Triple Negative Breast Cancer Therapy. Int. J. Nanomed..

[29] Si Y., Zhang Y., Ngo H.G., Guan J.-S., Chen K., Wang Q., Singh A.P., Xu Y., Zhou L., Yang E.S. (2021). Targeted Liposomal Chemotherapies to Treat Triple-Negative Breast Cancer. Cancers.

[30] Zeng W., Luo Y., Gan D., Zhang Y., Deng H., Liu G. (2023). Advances in Doxorubicin-based nano-drug delivery system in triple negative breast cancer. Front. Bioeng. Biotechnol..

[31] Jawad M., Öztürk K., Jabir M.S. (2021). TNF-α loaded on gold nanoparticles as promising drug delivery system against proliferation of breast cancer cells. Mater. Today Proc..

[32] Zhou B., Xue J., Wu R., Meng H., Li R., Mo Z., Zhai H., Chen X., Liu R., Lai G. (2023). CREBZF mRNA nanoparticles suppress breast cancer progression through a positive feedback loop boosted by circPAPD4. J. Exp. Clin. Cancer Res..

[33] Ji P., Wang X., Yin J., Mou Y., Huang H., Ren Z. (2022). Selective delivery of curcumin to breast cancer cells by self-targeting apoferritin nanocages with pH-responsive and low toxicity. Drug Deliv..

[34] Guo Z., Gao X., Lu J., Li Y., Jin Z., Fahad A., Pambe N.U., Ejima H., Sun X., Wang X. (2024). Apoptosis and Paraptosis Induced by Disulfiram-Loaded Ca^2+^/Cu^2+^ Dual-Ions Nano Trap for Breast Cancer Treatment. ACS Nano.

[35] Menghini S., Vizovisek M., Enders J., Schuerle S. (2023). Magnetospirillum magneticum triggers apoptotic pathways in human breast cancer cells. Cancer Metab..

[36] Abdellatif A.A., Alsharidah M., Al Rugaie O., Tawfeek H.M., Tolba N.S. (2021). Silver Nanoparticle-Coated Ethyl Cellulose Inhibits Tumor Necrosis Factor-α of Breast Cancer Cells. Drug Des. Dev. Ther..

[37] Chaudhari R., Nasra S., Meghani N., Kumar A. (2022). MiR-206 conjugated gold nanoparticle based targeted therapy in breast cancer cells. Sci. Rep..

[38] Naderi S., Zare H., Taghavinia N., Irajizad A., Aghaei M., Panjehpour M. (2018). Cadmium telluride quantum dots induce apoptosis in human breast cancer cell lines. Toxicol. Ind. Heal..

[39] Seyedi S.M.R., Asoodeh A., Darroudi M. (2022). The human immune cell simulated anti-breast cancer nanorobot: The efficient, traceable, and dirigible anticancer bio-bot. Cancer Nanotechnol..

[40] Yu X., Li X., Chen Q., Wang S., Xu R., He Y., Qin X., Zhang J., Yang W., Shi L. (2024). High Intensity Focused Ultrasound-Driven Nanomotor for Effective Ferroptosis-Immunotherapy of TNBC. Adv. Sci..

[41] Wu W., Yang Y., Liang Z., Song X., Huang Y., Qiu L., Qiu X., Yu S., Xue W. (2021). Near infrared II laser controlled free radical releasing nanogenerator for synergistic nitric oxide and alkyl radical therapy of breast cancer. Nanoscale.

[42] Wang C., Li T., Wang Z., Li Y., Liu Y., Xu M., Zhang Z., Deng Y., Cai L., Zhang C. (2023). Nano-modulators with the function of disrupting mitochondrial Ca2+ homeostasis and photothermal conversion for synergistic breast cancer therapy. J. Nanobiotechnol..

[43] Nunes T., Pons T., Hou X., Van Do K., Caron B., Rigal M., Di Benedetto M., Palpant B., Leboeuf C., Janin A. (2019). Pulsed-laser irradiation of multifunctional gold nanoshells to overcome trastuzumab resistance in HER2-overexpressing breast cancer. J. Exp. Clin. Cancer Res..

[44] Yang T., Yao Q., Cao F., Liu Q., Liu B., Wang X.-H. (2016). Silver nanoparticles inhibit the function of hypoxia-inducible factor-1 and target genes: Insight into the cytotoxicity and antiangiogenesis. Int. J. Nanomed..

[45] Guo P., Yang J., Jia D., Moses M.A., Auguste D.T. (2016). ICAM-1-Targeted, Lcn2 siRNA-Encapsulating Liposomes are Potent Anti-angiogenic Agents for Triple Negative Breast Cancer. Theranostics.

[46] Sun K., Yu W., Ji B., Chen C., Yang H., Du Y., Song M., Cai H., Yan F., Su R. (2019). Saikosaponin D loaded macrophage membrane-biomimetic nanoparticles target angiogenic signaling for breast cancer therapy. Appl. Mater. Today.

[47] Ye H., Wang K., Lu Q., Zhao J., Wang M., Kan Q., Zhang H., Wang Y., He Z., Sun J. (2020). Nanosponges of circulating tumor-derived exosomes for breast cancer metastasis inhibition. Biomaterials.

[48] Wang J., Xu H., Li S., Lin M., Lu Y., Liu K., Katanaev V., Denisov E.V., Jia L. (2023). Long cruising aptamer-albumin nanobots intelligently capture and restrain circulating tumor cells. Nano Today.

[49] Zhai Y., Ran W., Su J., Lang T., Meng J., Wang G., Zhang P., Li Y. (2018). Traceable Bioinspired Nanoparticle for the Treatment of Metastatic Breast Cancer via NIR-Trigged Intracellular Delivery of Methylene Blue and Cisplatin. Adv. Mater..

[50] Olmos S.P., Torres R.D., Elbakrawy E., Hughes L., Mckenna J., Hill M.A., Kadhim M., Noguera P.R., Bolanos-Garcia V.M. (2019). Combinatorial Use of Chitosan Nanoparticles, Reversine, and Ionising Radiation on Breast Cancer Cells Associated with Mitosis Deregulation. Biomolecules.

[51] Kang T.-W., Kim H.-S., Lee B.-C., Shin T.-H., Choi S.W., Kim Y.-J., Lee H.-Y., Jung Y.-K., Seo K.-W., Kang K.-S. (2015). Mica Nanoparticle, STB-HO Eliminates the Human Breast Carcinoma Cells by Regulating the Interaction of Tumor with its Immune Microenvironment. Sci. Rep..

[52] Ma W., Zhang Y., Shao X., Xie X., Mao C., Cui W., Li Q., Shi J., Li J., Fan C. (2019). An Intelligent DNA Nanorobot with *in Vitro* Enhanced Protein Lysosomal Degradation of HER2. Nano Lett..

[53] Lewińska A., Radoń A., Gil K., Błoniarz D., Ciuraszkiewicz A., Kubacki J., Kądziołka-Gaweł M., Łukowiec D., Gębara P., Krogul-Sobczak A. (2024). Carbon-Coated Iron Oxide Nanoparticles Promote Reductive Stress-Mediated Cytotoxic Autophagy in Drug-Induced Senescent Breast Cancer Cells. ACS Appl. Mater. Interfaces.

[54] Valent P., Groner B., Schumacher U., Superti-Furga G., Busslinger M., Kralovics R., Zielinski C., Penninger J.M., Kerjaschki D., Stingl G. (2016). Paul Ehrlich (1854-1915) and His Contributions to the Foundation and Birth of Translational Medicine. J. Innate Immun..

[55] Blair J. (2017). Making magic bullets. Nat. Microbiol..

[56] Tewabe A., Abate A., Tamrie M., Seyfu A., Siraj E.A. (2021). Targeted Drug Delivery—From Magic Bullet to Nanomedicine: Principles, Challenges, and Future Perspectives. J. Multidiscip. Heal..

[57] Pumera M. (2010). Electrochemically powered self-propelled electrophoretic nanosubmarines. Nanoscale.

[58] Xu Z., Ni R., Chen Y. (2019). Targeting breast cancer stem cells by a self-assembled, aptamer-conjugated DNA nanotrain with preloading doxorubicin. Int. J. Nanomed..

[59] Hameed M.K., Parambath J.B.M., Gul M.T., Khan A.A., Park Y., Han C., Mohamed A.A. (2022). Arylated gold nanostars aided SERS study of breast cancer cells. Appl. Surf. Sci..

[60] Fatima S.W., Imtiyaz K., Alam Rizvi M.M., Khare S.K. (2021). Microbial transglutaminase nanoflowers as an alternative nanomedicine for breast cancer theranostics. RSC Adv..

[61] Patel H., Parekh K., Gamarra L.F., Mamani J.B., Alves A.d.H., Neto A.F. (2023). In vitro evaluation of magnetic fluid hyperthermia therapy on breast cancer cells using monodispersed Mn0.5Zn0.5Fe2O4 nanoflowers. J. Magn. Magn. Mater..

[62] Liang L., Huo W., Wang B., Cao L., Huo H., Liu Y., Jin Y., Yang X. (2021). DNAzyme-Based nanoflowers for reversing P-glycoprotein-mediated multidrug resistance in breast cancer. J. Colloid Interface Sci..

[63] Cao H., Li C., Qi W., Meng X., Tian R., Qi Y., Yang W., Li J. (2017). Synthesis, cytotoxicity and antitumour mechanism investigations of polyoxometalate doped silica nanospheres on breast cancer MCF-7 cells. PLOS ONE.

[64] Arjama M., Mehnath S., Jeyaraj M. (2022). Self-assembled hydrogel nanocube for stimuli responsive drug delivery and tumor ablation by phototherapy against breast cancer. Int. J. Biol. Macromol..

[65] White B.E., White M.K., Alsudani Z.A.N., Watanabe F., Biris A.S., Ali N. (2022). Cellular Uptake of Gold Nanorods in Breast Cancer Cell Lines. Nanomaterials.

[66] Yin Y., Hao Y., Wang N., Yang P., Li N., Zhang X., Song Y., Feng X., Ma W. (2020). PPy nanoneedle based nanoplatform capable of overcoming biological barriers for synergistic chemo-photothermal therapy. RSC Adv..

[67] Safarkhani M., Moghaddam S.S., Taghavimandi F., Bagherzadeh M., Fatahi Y., Park U., Radmanesh F., Huh Y.S., Rabiee N. (2023). Bioengineered Smart Nanocarriers for Breast Cancer Treatment: Adorned Carbon-Based Nanocomposites with Silver and Palladium Complexes for Efficient Drug Delivery. ACS Omega.

[68] Peng J., Chen J., Xie F., Bao W., Xu H., Wang H., Xu Y., Du Z. (2019). Herceptin-conjugated paclitaxel loaded PCL-PEG worm-like nanocrystal micelles for the combinatorial treatment of HER2-positive breast cancer. Biomaterials.

[69] Sun S., Liu Y., He C., Hu W., Liu W., Huang X., Wu J., Xie F., Chen C., Wang J. (2021). Combining NanoKnife with M1 oncolytic virus enhances anticancer activity in pancreatic cancer. Cancer Lett..

[70] Xie B., Yang X., Zhang R., Guo J., Chen Z., He Y. (2021). Hollow and Porous Fe3C-NC Nanoballoons Nanozymes for Cancer Cell H2O2 Detection. Sensors Actuators B Chem..

[71] Zhang X., Liu Y., Doungchawee J., Castellanos-García L.J., Sikora K.N., Jeon T., Goswami R., Fedeli S., Gupta A., Huang R. (2023). Bioorthogonal nanozymes for breast cancer imaging and therapy. J. Control. Release.

[72] Dehariya D., Eswar K., Tarafdar A., Balusamy S., Rengan A.K. (2023). Recent advances of nanobubble-based systems in cancer therapeutics: A Review. Biomed. Eng. Adv..

[73] Jugniot N., Massoud T.F., Dahl J.J., Paulmurugan R. (2022). Biomimetic nanobubbles for triple-negative breast cancer targeted ultrasound molecular imaging. J. Nanobiotechnol..

[74] Ombredane A.S., Araujo V.H., Borges C.O., Costa P.L., Landim M.G., Pinheiro A.C., Szlachetka O., Benedito L.E., Espindola L.S., Dias D.J. (2020). Nanoemulsion-based systems as a promising approach for enhancing the antitumoral activity of pequi oil (Caryocar brasilense Cambess.) in breast cancer cells. J. Drug Deliv. Sci. Technol..

[75] Bakherad H., Ghasemi F., Hosseindokht M., Zare H. (2022). Nanobodies; new molecular instruments with special specifications for targeting, diagnosis and treatment of triple-negative breast cancer. Cancer Cell Int..

[76] Gajdosova V., Lorencova L., Kasak P., Tkac J. (2020). Electrochemical Nanobiosensors for Detection of Breast Cancer Biomarkers. Sensors.

[77] Zhang L., Burns N., Ji Z., Sun S., Deutscher S.L., Carson W.E., Guo P. (2023). Nipple fluid for breast cancer diagnosis using the nanopore of Phi29 DNA-packaging motor. Nanomed. Nanotechnol. Biol. Med..

[78] Si P., Yu W., Li C., Chen H., Zhang E., Gu J., Wang R., Shi J. (2023). Oxygen-independent alkyl radical nanogenerator enhances breast cancer therapy. Nanomed. Nanotechnol. Biol. Med..

[79] Bahreyni A., Mohamud Y., Luo H. (2020). Emerging nanomedicines for effective breast cancer immunotherapy. J. Nanobiotechnol..

[80] Zhang X., Galenkamp N.S., van der Heide N.J., Moreno J., Maglia G., Kjems J. (2023). Specific Detection of Proteins by a Nanobody-Functionalized Nanopore Sensor. ACS Nano.

[81] Neagu A.-N., Whitham D., Bruno P., Morrissiey H., Darie C.A., Darie C.C. (2023). Omics-Based Investigations of Breast Cancer. Molecules.

[82] Tiambeng T.N., Roberts D.S., Brown K.A., Zhu Y., Chen B., Wu Z., Mitchell S.D., Guardado-Alvarez T.M., Jin S., Ge Y. (2020). Nanoproteomics enables proteoform-resolved analysis of low-abundance proteins in human serum. Nat. Commun..

[83] Zhang Y., Zhu M., Zhu J., Xu F., Chen Y. (2023). Nanoproteomics deciphers the prognostic value of EGFR family proteins-based liquid biopsy. Anal. Biochem..

[84] Liu J., Ding G., Chen S., Xue C., Chen M., Wu X., Yuan Q., Zheng J., Yang R. (2021). Multifunctional Programmable DNA Nanotrain for Activatable Hypoxia Imaging and Mitochondrion-Targeted Enhanced Photodynamic Therapy. ACS Appl. Mater. Interfaces.

[85] Minh T., Ncibi M., Srivastava V., Doshi B., Sillanpää M. (2021). Micro/nano-machines for spilled-oil cleanup and recovery: A review. Chemosphere.

[86] Chattha G.M., Arshad S., Kamal Y., Chattha M.A., Asim M.H., Raza S.A., Mahmood A., Manzoor M., Dar U.I., Arshad A. (2023). Nanorobots: An innovative approach for DNA-based cancer treatment. J. Drug Deliv. Sci. Technol..

[87] Rajendran S., Sundararajan P., Awasthi A., Rajendran S. (2024). Nanorobotics in Medicine: A Systematic Review of Advances, Challenges, and Future Prospects with a Focus on Cell Therapy, Invasive Surgery, and Drug Delivery. Precis. Nanomed..

[88] Aggarwal M., Kumar S. (2022). The Use of Nanorobotics in the Treatment Therapy of Cancer and Its Future Aspects: A Review. Cureus.

[89] Peng X., Tang S., Tang D., Zhou D., Li Y., Chen Q., Wan F., Lukas H., Han H., Zhang X. (2023). Autonomous metal-organic framework nanorobots for active mitochondria-targeted cancer therapy. Sci. Adv..

[90] Buchke S., Sharma M., Bora A., Relekar M., Bhanu P., Kumar J. (2022). Mitochondria-Targeted, Nanoparticle-Based Drug-Delivery Systems: Therapeutics for Mitochondrial Disorders. Life.

[91] Liu Y., Jia H.-R., Han X., Wu F.-G. (2021). Endoplasmic reticulum-targeting nanomedicines for cancer therapy. Smart Mater. Med..

[92] Milane L.S., Dolare S., Ren G., Amiji M. (2023). Combination Organelle Mitochondrial Endoplasmic Reticulum Therapy (COMET) for Multidrug Resistant Breast Cancer. J. Control. Release.

[93] Liu Q., Zhang X., Zhang J. (2022). Exosome-Based Nanoplatforms: The Emerging Tools for Breast Cancer Therapy. Front. Oncol..

[94] Hu J., Liu Y., Du Y., Peng X., Liu Z. (2023). Cellular organelles as drug carriers for disease treatment. J. Control. Release.

[95] Zhang D., Liu S., Guan J., Mou F. (2022). “Motile-targeting” drug delivery platforms based on micro/nanorobots for tumor therapy. Front. Bioeng. Biotechnol..

[96] Zhang S., Scott E.Y., Singh J., Chen Y., Zhang Y., Elsayed M., Chamberlain M.D., Shakiba N., Adams K., Yu S. (2019). The optoelectronic microrobot: A versatile toolbox for micromanipulation. Proc. Natl. Acad. Sci. USA.

[97] Zhou H., Mayorga-Martinez C.C., Pané S., Zhang L., Pumera M. (2021). Magnetically Driven Micro and Nanorobots. Chem. Rev..

[98] Wang Y., Chen J., Su G., Mei J., Li J. (2023). A Review of Single-Cell Microrobots: Classification, Driving Methods and Applications. Micromachines.

[99] Zhou M., Yin Y., Zhao J., Zhou M., Bai Y., Zhang P. (2023). Applications of microalga-powered microrobots in targeted drug delivery. Biomater. Sci..

[100] Singh A.V., Ansari M.H.D., Mahajan M., Srivastava S., Kashyap S., Dwivedi P., Pandit V., Katha U. (2020). Sperm Cell Driven Microrobots—Emerging Opportunities and Challenges for Biologically Inspired Robotic Design. Micromachines.

[101] Chen H., Zhou T., Li S., Feng J., Li W., Li L., Zhou X., Wang M., Li F., Zhao X. (2023). Living Magnetotactic Microrobots Based on Bacteria with a Surface-Displayed CRISPR/Cas12a System for Penaeus Viruses Detection. ACS Appl. Mater. Interfaces.

[102] Gwisai T., Mirkhani N., Christiansen M.G., Nguyen T.T., Ling V., Schuerle S. (2022). Magnetic torque–driven living microrobots for increased tumor infiltration. Sci. Robot..

[103] Huang H., Lang Y., Wang S., Zhou M. Microalgae-based drug delivery systems in biomedical applications. Eng. Regen..

[104] Shen H., Cai S., Wang Z., Ge Z., Yang W. (2023). Magnetically driven microrobots: Recent progress and future development. Mater. Des..

[105] Stanley S. (2014). Biological nanoparticles and their influence on organisms. Curr. Opin. Biotechnol..

[106] Kotakadi S.M., Borelli D.P.R., Nannepaga J.S. (2022). Therapeutic Applications of Magnetotactic Bacteria and Magnetosomes: A Review Emphasizing on the Cancer Treatment. Front. Bioeng. Biotechnol..

[107] Song X., Fu W., Cheang U.K. (2022). Immunomodulation and delivery of macrophages using nano-smooth drug-loaded magnetic microrobots for dual targeting cancer therapy. iScience.

[108] Hanahan D., Weinberg R.A. (2000). The Hallmarks of Cancer. Cell.

[109] Hanahan D., Weinberg R.A. (2011). Hallmarks of cancer: The next generation. Cell.

[110] Hanahan D. (2022). Hallmarks of Cancer: New Dimensions. Cancer Discov..

[111] Naser R., Fakhoury I., El-Fouani A., Abi-Habib R., El-Sibai M. (2022). Role of the tumor microenvironment in cancer hallmarks and targeted therapy (Review). Int. J. Oncol..

[112] Huang T., Song X., Yang Y., Wan X., Alvarez A.A., Sastry N., Feng H., Hu B., Cheng S.-Y. (2018). Autophagy and Hallmarks of Cancer. Crit. Rev. Oncog..

[113] Saha T., Solomon J., Samson A.O., Gil-Henn H. (2021). Invasion and Metastasis as a Central Hallmark of Breast Cancer. J. Clin. Med..

[114] Zhou Y., Yuan J., Xu K., Li S., Liu Y. (2024). Nanotechnology Reprogramming Metabolism for Enhanced Tumor Immunotherapy. ACS Nano.

[115] Feitelson M.A., Arzumanyan A., Kulathinal R.J., Blain S.W., Holcombe R.F., Mahajna J., Marino M., Martinez-Chantar M.L., Nawroth R., Sanchez-Garcia I. (2015). Sustained proliferation in cancer: Mechanisms and novel therapeutic targets. Semin. Cancer Biol..

[116] Wu X., Wu M.-Y., Jiang M., Zhi Q., Bian X., Xu M.-D., Gong F.-R., Hou J., Tao M., Shou L.-M. (2017). TNF-α sensitizes chemotherapy and radiotherapy against breast cancer cells. Cancer Cell Int..

[117] Yalamarty S.S.K., Filipczak N., Khan M.M., Torchilin V.P. (2023). Role of circular RNA and its delivery strategies to cancer – An overview. J. Control. Release.

[118] Amin A.R., Karpowicz P.A., Carey T.E., Arbiser J., Nahta R., Chen Z.G., Dong J.-T., Kucuk O., Khan G.N., Huang G.S. (2015). Evasion of anti-growth signaling: A key step in tumorigenesis and potential target for treatment and prophylaxis by natural compounds. Semin. Cancer Biol..

[119] Sun L., Liu Y., Yang N., Ye X., Liu Z., Wu J., Zhou M., Zhong W., Cao M., Zhang J. (2023). Gold nanoparticles inhibit tumor growth via targeting the Warburg effect in a c-Myc-dependent way. Acta Biomater..

[120] Zhang Z., Yao Y., Yuan Q., Lu C., Zhang X., Yuan J., Hou K., Zhang C., Du Z., Gao X. (2020). Gold clusters prevent breast cancer bone metastasis by suppressing tumor-induced osteoclastogenesis. Theranostics.

[121] Nel J., Elkhoury K., Velot É., Bianchi A., Acherar S., Francius G., Tamayol A., Grandemange S., Arab-Tehrany E. (2023). Functionalized liposomes for targeted breast cancer drug delivery. Bioact. Mater..

[122] Yu J., Xu J., Jiang R., Yuan Q., Ding Y., Ren J., Jiang D., Wang Y., Wang L., Chen P. (2024). Versatile chondroitin sulfate-based nanoplatform for chemo-photodynamic therapy against triple-negative breast cancer. Int. J. Biol. Macromol..

[123] Yuan L., Cai Y., Zhang L., Liu S., Li P., Li X. (2022). Promoting Apoptosis, a Promising Way to Treat Breast Cancer With Natural Products: A Comprehensive Review. Front. Pharmacol..

[124] Hayes J.D., Dinkova-Kostova A.T., Tew K.D. (2020). Oxidative Stress in Cancer. Cancer Cell.

[125] Bhaskar S., Lim S. (2017). Engineering protein nanocages as carriers for biomedical applications. NPG Asia Mater..

[126] Dong X., Li Y., Sheng X., Zhou W., Sun A., Dai H. (2024). Mitochondria-related signaling pathways involved in breast cancer regulate ferroptosis. Genes Dis..

[127] Liu N., Chen M. (2024). Crosstalk between ferroptosis and cuproptosis: From mechanism to potential clinical application. Biomed. Pharmacother..

[128] Xu C., Chen Y., Yu Q., Song J., Jin Y., Gao X. (2023). Compounds targeting ferroptosis in breast cancer: Progress and their therapeutic potential. Front. Pharmacol..

[129] Ko M.J., Min S., Hong H., Yoo W., Joo J., Zhang Y.S., Kang H., Kim D.-H. (2024). Magnetic nanoparticles for ferroptosis cancer therapy with diagnostic imaging. Bioact. Mater..

[130] Xu C.-C., Lin Y.-F., Huang M.-Y., Zhang X.-L., Wang P., Huang M.-Q., Lu J.-J. (2023). Paraptosis: A non-classical paradigm of cell death for cancer therapy. Acta Pharmacol. Sin..

[131] Li Y., Li Z. (2021). Potential Mechanism Underlying the Role of Mitochondria in Breast Cancer Drug Resistance and Its Related Treatment Prospects. Front. Oncol..

[132] Zhang Y., Zhang H., Wang M., Schmid T., Xin Z., Kozhuharova L., Yu W.-K., Huang Y., Cai F., Biskup E. (2021). Hypoxia in Breast Cancer—Scientific Translation to Therapeutic and Diagnostic Clinical Applications. Front. Oncol..

[133] Sadlecki P., Bodnar M., Grabiec M., Marszalek A., Walentowicz P., Sokup A., Zegarska J., Walentowicz-Sadlecka M. (2014). The Role of Hypoxia-Inducible Factor-1*α*, Glucose Transporter-1, (GLUT-1) and Carbon Anhydrase IX in Endometrial Cancer Patients. BioMed Res. Int..

[134] Kurihara T., Westenskow P.D., Friedlander M. (2014). Hypoxia-Inducible Factor (HIF)/Vascular Endothelial Growth Factor (VEGF) Signaling in the Retina. Adv. Exp. Med. Biol.

[135] Garg A., Lai W.-C., Chopra H., Agrawal R., Singh T., Chaudhary R., Dubey B.N. (2024). Nanosponge: A promising and intriguing strategy in medical and pharmaceutical Science. Heliyon.

[136] Tiwari K., Bhattacharya S. (2022). The ascension of nanosponges as a drug delivery carrier: Preparation, characterization, and applications. J. Mater. Sci. Mater. Med..

[137] Iravani S., Varma R.S. (2022). Nanosponges for Drug Delivery and Cancer Therapy: Recent Advances. Nanomaterials.

[138] Zhu L., Yu X., Cao T., Deng H., Tang X., Lin Q., Zhou Q. (2023). Immune cell membrane-based biomimetic nanomedicine for treating cancer metastasis. Acta Pharm. Sin. B.

[139] Garanina A.S., Vishnevskiy D.A., Chernysheva A.A., Valikhov M.P., Malinovskaya J.A., Lazareva P.A., Semkina A.S., Abakumov M.A., Naumenko V.A. (2023). Neutrophil as a Carrier for Cancer Nanotherapeutics: A Comparative Study of Liposome, PLGA, and Magnetic Nanoparticles Delivery to Tumors. Pharmaceuticals.

[140] Zhang Q., Dehaini D., Zhang Y., Zhou J., Chen X., Zhang L., Fang R.H., Gao W., Zhang L. (2018). Neutrophil membrane-coated nanoparticles inhibit synovial inflammation and alleviate joint damage in inflammatory arthritis. Nat. Nanotechnol..

[141] Fang Z., Fang J., Gao C., Gao R., Lin P., Yu W. (2022). Recent trends in platelet membrane-cloaked nanoparticles for application of inflammatory diseases. Drug Deliv..

[142] Wavhale R.D., Dhobale K.D., Rahane C.S., Chate G.P., Tawade B.V., Patil Y.N., Gawade S.S., Banerjee S.S. (2021). Water-powered self-propelled magnetic nanobot for rapid and highly efficient capture of circulating tumor cells. Commun. Chem..

[143] Rasool R., Ullah I., Mubeen B., Alshehri S., Imam S.S., Ghoneim M.M., Alzarea S.I., Al-Abbasi F.A., Murtaza B.N., Kazmi I. (2022). Theranostic Interpolation of Genomic Instability in Breast Cancer. Int. J. Mol. Sci..

[144] Bielski C.M., Taylor B.S. (2021). Homing in on genomic instability as a therapeutic target in cancer. Nat. Commun..

[145] McAinsh A.D., Kops G.J.P.L. (2023). Principles and dynamics of spindle assembly checkpoint signalling. Nat. Rev. Mol. Cell Biol..

[146] Prajumwongs P., Waenphimai O., Vaeteewoottacharn K., Wongkham S., Sawanyawisuth K. (2021). Reversine, a selective MPS1 inhibitor, induced autophagic cell death via diminished glucose uptake and ATP production in cholangiocarcinoma cells. PeerJ.

[147] Wang M., Zhang C., Song Y., Wang Z., Wang Y., Luo F., Xu Y., Zhao Y., Wu Z., Xu Y. (2017). Mechanism of immune evasion in breast cancer. OncoTargets Ther..

[148] Wang S., Wang J., Chen Z., Luo J., Guo W., Sun L., Lin L. (2024). Targeting M2-like tumor-associated macrophages is a potential therapeutic approach to overcome antitumor drug resistance. npj Precis. Oncol..

[149] D’Avanzo N., Torrieri G., Figueiredo P., Celia C., Paolino D., Correia A., Moslova K., Teesalu T., Fresta M., Santos H.A. (2021). LinTT1 peptide-functionalized liposomes for targeted breast cancer therapy. Int. J. Pharm..

[150] Li Q., Ming R., Huang L., Zhang R. (2024). Versatile Peptide-Based Nanosystems for Photodynamic Therapy. Pharmaceutics.

[151] Mas-Bargues C., Sanz-Ros J., Román-Domínguez A., Inglés M., Gimeno-Mallench L., El Alami M., Viña-Almunia J., Gambini J., Viña J., Borrás C. (2019). Relevance of Oxygen Concentration in Stem Cell Culture for Regenerative Medicine. Int. J. Mol. Sci..

[152] Chen Z., Han F., Du Y., Shi H., Zhou W. (2023). Hypoxic microenvironment in cancer: Molecular mechanisms and therapeutic interventions. Signal Transduct. Target. Ther..

[153] Zhang J., Tang K., Fang R., Liu J., Liu M., Ma J., Wang H., Ding M., Wang X., Song Y. (2023). Nanotechnological strategies to increase the oxygen content of the tumor. Front. Pharmacol..

[154] Martel S., Mohammadi M. (2016). Switching between Magnetotactic and Aerotactic Displacement Controls to Enhance the Efficacy of MC-1 Magneto-Aerotactic Bacteria as Cancer-Fighting Nanorobots. Micromachines.

[155] Jiang W., Liang M., Lei Q., Li G., Wu S. (2023). The Current Status of Photodynamic Therapy in Cancer Treatment. Cancers.

[156] Fang H., Gai Y., Wang S., Liu Q., Zhang X., Ye M., Tan J., Long Y., Wang K., Zhang Y. (2021). Biomimetic oxygen delivery nanoparticles for enhancing photodynamic therapy in triple-negative breast cancer. J. Nanobiotechnol..

[157] Gao M., Liang C., Song X., Chen Q., Jin Q., Wang C., Liu Z. (2017). Erythrocyte-Membrane-Enveloped Perfluorocarbon as Nanoscale Artificial Red Blood Cells to Relieve Tumor Hypoxia and Enhance Cancer Radiotherapy. Adv. Mater..

[158] Wu Q., Sharma D. (2023). Autophagy and Breast Cancer: Connected in Growth, Progression, and Therapy. Cells.

[159] López-Méndez T.B., Sánchez-Álvarez M., Trionfetti F., Pedraz J.L., Tripodi M., Cordani M., Strippoli R., González-Valdivieso J. (2023). Nanomedicine for autophagy modulation in cancer therapy: A clinical perspective. Cell Biosci..

[160] Laha D., Pramanik A., Maity J., Mukherjee A., Pramanik P., Laskar A., Karmakar P. (2014). Interplay between autophagy and apoptosis mediated by copper oxide nanoparticles in human breast cancer cells MCF7. Biochim. et Biophys. Acta (BBA) Gen. Subj..

[161] Horimoto Y., Ishizuka Y., Ueki Y., Higuchi T., Arakawa A., Saito M. (2022). Comparison of tumors with HER2 overexpression versus HER2 amplification in HER2-positive breast cancer patients. BMC Cancer.

